# A Risk Prediction Model for Physical Restraints Among Older Chinese Adults in Long-term Care Facilities: Machine Learning Study

**DOI:** 10.2196/43815

**Published:** 2023-04-06

**Authors:** Jun Wang, Hongmei Chen, Houwei Wang, Weichu Liu, Daomei Peng, Qinghua Zhao, Mingzhao Xiao

**Affiliations:** 1 Department of Nursing The First Affiliated Hospital of Chongqing Medical University Chongqing China; 2 Department of Gynecology The First Affiliated Hospital of Chongqing Medical University Chongqing China; 3 College of Mathematics and Physics Chongqing University of Science and Technology Chongqing China; 4 Aged Care Unit The First Social Welfare Home of Chongqing Chongqing China; 5 Department of Urology The First Affiliated Hospital of Chongqing Medical University Chongqing China

**Keywords:** physical restraint, prediction model, machine learning, stacking ensemble model, model, older adults, elderly, risk factor, learning model, development, support, accuracy, precision, cognitive impairment, utility, management

## Abstract

**Background:**

Numerous studies have identified risk factors for physical restraint (PR) use in older adults in long-term care facilities. Nevertheless, there is a lack of predictive tools to identify high-risk individuals.

**Objective:**

We aimed to develop machine learning (ML)–based models to predict the risk of PR in older adults.

**Methods:**

This study conducted a cross-sectional secondary data analysis based on 1026 older adults from 6 long-term care facilities in Chongqing, China, from July 2019 to November 2019. The primary outcome was the use of PR (yes or no), identified by 2 collectors’ direct observation. A total of 15 candidate predictors (older adults’ demographic and clinical factors) that could be commonly and easily collected from clinical practice were used to build 9 independent ML models: Gaussian Naïve Bayesian (GNB), k-nearest neighbor (KNN), decision tree (DT), logistic regression (LR), support vector machine (SVM), random forest (RF), multilayer perceptron (MLP), extreme gradient boosting (XGBoost), and light gradient boosting machine (Lightgbm), as well as stacking ensemble ML. Performance was evaluated using accuracy, precision, recall, an *F* score, a comprehensive evaluation indicator (CEI) weighed by the above indicators, and the area under the receiver operating characteristic curve (AUC). A net benefit approach using the decision curve analysis (DCA) was performed to evaluate the clinical utility of the best model. Models were tested via 10-fold cross-validation. Feature importance was interpreted using Shapley Additive Explanations (SHAP).

**Results:**

A total of 1026 older adults (mean 83.5, SD 7.6 years; n=586, 57.1% male older adults) and 265 restrained older adults were included in the study. All ML models performed well, with an AUC above 0.905 and an *F* score above 0.900. The 2 best independent models are RF (AUC 0.938, 95% CI 0.914-0.947) and SVM (AUC 0.949, 95% CI 0.911-0.953). The DCA demonstrated that the RF model displayed better clinical utility than other models. The stacking model combined with SVM, RF, and MLP performed best with AUC (0.950) and CEI (0.943) values, as well as the DCA curve indicated the best clinical utility. The SHAP plots demonstrated that the significant contributors to model performance were related to cognitive impairment, care dependency, mobility decline, physical agitation, and an indwelling tube.

**Conclusions:**

The RF and stacking models had high performance and clinical utility. ML prediction models for predicting the probability of PR in older adults could offer clinical screening and decision support, which could help medical staff in the early identification and PR management of older adults.

## Introduction

Physical restraint (PR) is not only an important indicator to measure the quality of medical care but is also a major public health issue that has aroused widespread concern worldwide. PR is defined as “any action or procedure that prevents a person’s free body movement to a position of choice or normal access to his or her body by the use of any method, attached or adjacent to a person’s body that he or she cannot control or remove easily” [1]. It is usually used to protect patients from falls, self-extubation, or injuries in intensive care units or psychiatric hospitals. Recent studies have shown that PR is more widely applied among older adults and results in worse outcomes. Older adults are 3 times more likely to endure PR than young people during hospitalization [2]. A systematic review and meta-analysis indicated that the pooled prevalence of PR among older adults in long-term care (LTC) facilities ranges from 22% (in North America) to 65% (in Australia) [3]. In China, the prevalence of PR among older adults in LTC was 62% in Taiwan [4], 52.7%-70.2% in Hong Kong [5], and 25.8% in mainland China (Chongqing) [6].

The World Health Organization has reported that restraining individuals could be considered maltreatment [7]. As older adults are vulnerable to various health-related problems, PR is used more frequently and lasts longer, resulting in more serious injuries. Previous studies have demonstrated that PR is inadequate for protection [8-10]. Conversely, PR is associated with negative consequences on physical (eg, pressure ulcers, fractures, and urinary and fecal incontinence), psychological (eg, cognitive decline, depression, anxiety, aggression, and fear), and social functions (eg, social isolation and loss of social worth), and even death [11-16]. Therefore, the early identification of high-risk individuals and early interventions are of great significance in preventing PR use, which can reduce the negative impact on health, society, and the economy.

Numerous studies have identified that the risk factors of PR use are associated with (1) individual-related factors (eg, age, cognitive impairment, mobility decline, care dependency, fall risk, etc) [16-18], (2) facility-related factors (eg, facility type, ownership, and staff levels) [6,17,19], and (3) caregiver-related factors (eg, knowledge, attitude, and intention) [20-22]. Nevertheless, the interaction between these predictors and their clinical value remains unclear. Moreover, predictors of PR use are mainly determined using traditional statistical methods, with the limitations of difficulty dealing with high-dimensional data, nonlinear variables, and heterogeneous distribution [23,24]. Importantly, rare tools, such as the model proposed in this paper, can comprehensively assess restraint risks and support decision-making for staff and families. The clinical prediction model provides a new horizon. This would allow early detection and increased surveillance of at-risk older adults and the development of early targeted interventions for preventing and reducing PR.

Recently, machine learning (ML) algorithms such as Naïve Bayes and random forest (RF) have been used in various fields for clinical practice such as diagnosis, occurrence, and prognosis [25-27]. Various robust ML prediction models have been developed for adverse events and complications prediction, such as cognitive impairment prediction [28], falls prediction [29], pressure injury prediction [30], and delirium prediction [31]. Current regular risk prediction models were developed using a generalized linear model, which depends on the implicit assumption that each risk factor is related in a linear fashion to outcomes. Although such a model is easy to code and fast to calculate, it may oversimplify the complex nonlinear interaction between variables. ML methods have been an alternative method to address current limitations. ML helps in handling information based on causal or statistical data, potentially revealing hidden dependencies between factors and diseases, and supporting clinical decisions [32]. To our knowledge, the development of a PR prediction model using ML has only been studied in psychiatric inpatients, limiting its predictive performance and application to broader scenarios [33,34]. Moreover, there are few studies on PR prediction among older adults in LTC facilities.

Given that our previous multicenter investigative study identified risk factors for PR, we have now proceeded to develop a prediction model for PR with information that can easily be provided by older adults in LTC facilities. The purpose of this study was to (1) develop and compare 9 independent ML models, (2) analyze the most important features of the 2 models with the best prediction performance, and (3) train and validate a more stable and generalized stacked model using the stacking ensemble learning algorithm.

## Methods

### Study Design and Participants

This study comprises a secondary analysis of multicenter cross-sectional data from 6 LTC facilities (ie, 1 aged-care center, 1 social welfare home, and 4 nursing homes) in Chongqing, China, from July 2019 to November 2019. Based on the inclusion criterion, all older adults who were present in the LTC facilities on the days of data collection were approached. The exclusion criteria were as follows: (1) older adults who have lived in LTC facilities for less than 2 weeks and (2) older adults whom we were not allowed to observe because of serious and special illnesses, brain death, or no voluntary movement ability. This study followed the transparent reporting of a multivariable prediction model for individual prognosis or diagnosis reporting guidelines [35].

### Ethics Approval

The study was approved by the Ethics Committee of the First Affiliated Hospital of Chongqing Medical University (approval number: 2019-104). Informed consent was obtained from all participants in the primary data collection, and the original informed consent allows the secondary analysis without additional consent. All research data are anonymous or deidentified to protect the privacy of participants. We did not provide any remuneration to each participant who provided complete and valid responses.

### Outcome and Predictors

The primary outcome was PR use (yes or no), gathered by 2 collectors through 3 direct observations, which was reported to be the most reliable method for collecting data on PR use [36]. The 3 separate observations were conducted on a working day at times when older adults were either most likely to be active or at rest. A “yes” response was recorded if at least one PR was used during the 3 data collection periods. The definition of PR complied with an international consensus [1]. We excluded bed rails as a means of PR because bed rails are conventionally pulled up in these facilities when older adults are lying in bed, but they can be removed freely as needed.

Predictors were identified based on a literature review and the clinical knowledge of practitioners who worked in LTC facilities. Details of the measurement and data collection are available in a previously published article [6]. A total of 15 predictors related to individual factors were included in this study: (1) older adults’ sociodemographic characteristics (ie, sex, age, and length of residence at LTC); and (2) clinical factors such as functional status on present living, including chronic diseases, mental diseases, consciousness, cognitive function, mobility, degree of care dependency, physical agitation, verbal agitation, depression symptoms, fecal and urinary conditions, fall risk, and indwelling tube. The definition of each variable was presented in Table S1 in Multimedia Appendix 1.

### Statistical Analysis

#### Overview

The demographic characteristics of older adults were described using descriptive statistics, such as means,  SD, numbers, and percentages. The chi-square test was performed to compare predictive variables between the PR and non-PR groups. These analyses were performed using IBM’s SPSS, version 25.0. All statistical tests were 2-sided, and a *P* value less than .05 was regarded as statistically significant.

**Figure 1:**
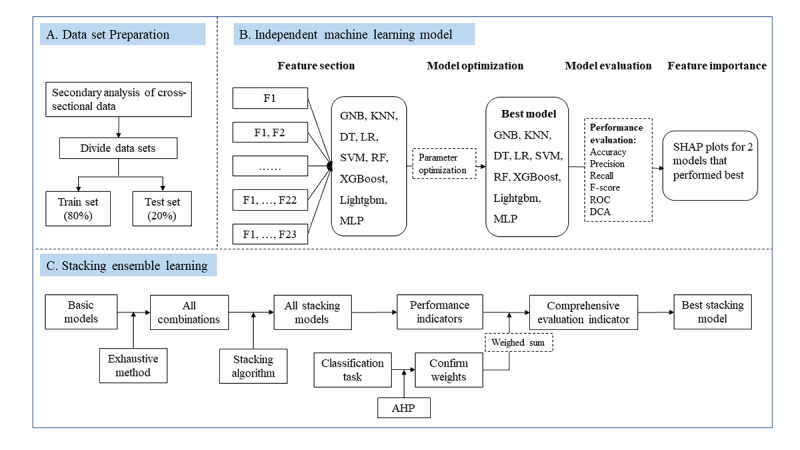
Flowchart summary of our methodology. AHP: analytic hierarchy process; DCA: decision curve analysis; DT: decision tree; GNB: Gaussian Naïve Bayesian; KNN: k-nearest neighbor; Lightgbm: light gradient boosting machine; LR: logistic regression; MLP: multilayer perceptron; RF: random forest; ROC: receiver operating characteristic; SHAP: Shapley Additive Explanations; SVM: support vector machine; XGBoost: extreme gradient boosting.

For the ML model development, we used a 2-step systematic framework comprising 9 widely applied independent ML methods and stacked ensemble-based ML models. Nine independent ML methods were used: logistic regression (LR), Gaussian Naïve Bayes (GNB), k-nearest neighbors (KNN), support vector machine (SVM), decision tree (DT), RF, extreme gradient boosting (XGBoost), light gradient boosting machine (Lightgbm), and multilayer perceptron (MLP). ML algorithms were performed in Python (version 3.7.4; Guido van Rossum). An overall flowchart of the analyses is shown in Figure 1.

This study was a secondary analysis of cross-sectional data that assessed the risk of PR without missing values or outliers. For data preparation, the data set was randomly divided into a training set (80%) and a test set (20%), and the samples were added with the same nonzero random seeds and stratified to ensure that the proportions of the cases in the training and test sets were equal and improved the stability of the model. The data set was split only once into a training set and a test set for each of the 9 independent ML models. The performance of the model was evaluated based on accuracy, precision, recall, *F* score, and area under the receiver operating characteristic curve (AUC; based on the test set), which were widely used in other studies [37-39]. The 95% CI of the AUC was calculated with bootstrapping, using 1000 iterations. Moreover, a decision curve analysis (DCA) was performed to evaluate the clinical utility of the 5 best performance prediction models by quantifying the net benefits.

#### Independent ML Model Development

For each model, we ranked feature importance based on RF and subsequently entered 15 features in order of importance using the iterative screening method. The model performance for each feature combination was then recorded. The final feature selection was obtained based on the optimal number of features and accuracy. After feature selection, the hyperparameters of the model were optimized using the Python sklearn (David Cournapeau) cross-validated grid search function. Specifically, the parameters tuned in each model were searched and optimized individually. Iterative tuning was performed for each parameter within the parameter range, and a visualized learning curve was used to select the optimal parameter value to reach the local optimum. The above steps were repeated for each parameter tuned in the model, and the optimal range of the parameters in each model was determined. Finally, a grid search through 10-fold cross-validation was performed to ascertain the best parameter combination value of the model. The trained model was then validated on the test data set, with the output being evaluation indicators of the model’s performance. We test the results via 10-fold cross-validation in the training set to avoid overfitting and assess the stability of the models. Furthermore, we used Shapley Additive Explanations (SHAP) to interpret and visualize the impact of predictors on PR risk based on the 2 models that performed best [40].

#### Stacking Ensemble Learning

We then used a stacking algorithm that has been shown to perform better than boosting and bagging ensemble classification algorithms [41,42]. The stacking-based algorithms contain cross-validation, which is used to select optimal basic model parameters. In stacking-based models, we also test the results via 10-fold cross-validation in the training set to avoid overfitting and assess the stability of the models. The importance of the classifier performance evaluation indicators should also be different for different classification tasks. In this study, a comprehensive evaluation indicator (CEI) based on the purpose of the classification task was used to evaluate the stacking model’s performance. It is defined as the weighted sum of the accuracy, precision, recall, and *F* score [43]. The weight of each indicator was determined using an analytic hierarchy process (AHP) [44]. In this study, we used the weight value calculated by Sun and Chen [43] with the values of accuracy (0.061), precision (0.293), recall (0.182), and *F* score (0.463). The weight value was calculated in a clinical setting to predict the occurrence of diseases, which was consistent with the requirements of our study prediction task. The AHP-stacking algorithm can be divided into 3 steps [43]: (1) list all possible basic model combinations using the exhaustive method based on the basic independent models, subsequently developing stacking models and output performance indicators; (2) determine the weights of the model performance indicators using AHP, based on classification tasks, and calculate the CEI of all stacking models; and (3) generate all stacking models and rank them in order of performance (ie, CEI).

## Results

### Participants’ Demographics

A total of 1026 older adults in 6 LTC facilities were included, comprising 265 older adults in the restraint group and 761 older adults in the nonrestraint group. Figure S1 in Multimedia Appendix 1 presents a flowchart of participant selection. Overall, the mean age was 83.47 (SD 7.62; range 60-102) years. A total of 586 (57.12%) participants were women. Table S2 in Multimedia Appendix 1 presents a univariate analysis of older adults with and without PR. Predictors comprising age, length of residence at LTC, number of chronic diseases, consciousness, cognitive function, mobility, care dependency, physical agitation, verbal agitation, depression symptoms, fecal and urinary conditions, fall risk, and indwelling tube showed significant differences between the PR and non-PR groups.

### Model Evaluation and Performance

**Figure 2:**
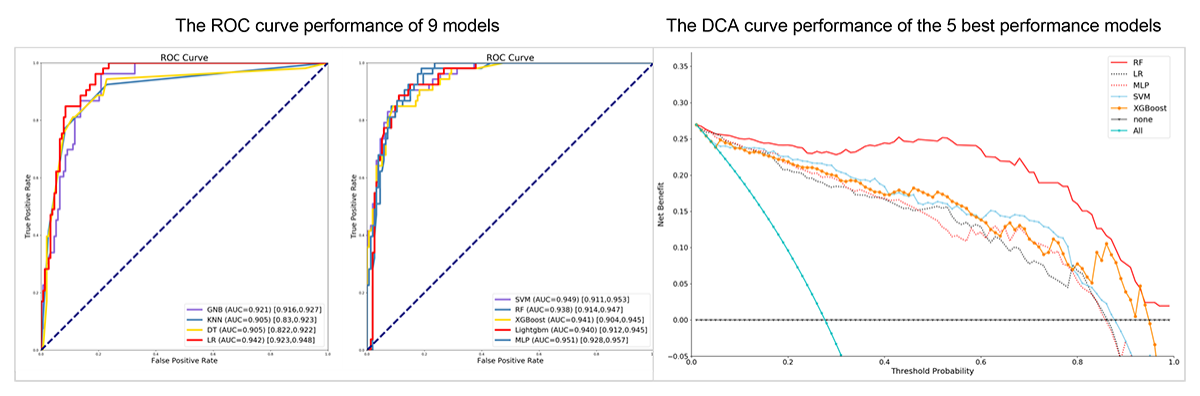
The ROC and DCA curves of the independent machine learning models. AUC: area under the receiver operating characteristic curve; DCA: decision curve analysis; DT: decision tree; GNB: Gaussian Naïve Bayesian; KNN: k-nearest neighbor; Lightgbm: light gradient boosting machine; LR: logistic regression; MLP: multilayer perceptron; RF: random forest; ROC: receiver operating characteristic; SVM: support vector machine; XGBoost: extreme gradient boosting.

The feature selection results showed that GNB performed best with 9 features, while the other models incorporated all features. Table 1 presents the predictive performances of the 9 models. Among them, the RF model performed with the highest accuracy (0.922), followed by the SVM (0.903). DT and GNB ranked the lowest (0.859). The RF model performed well with respect to precision (0.953), followed by GNB (0.949), MLP (0.939), SVM (0.929), and others above 0.900. The SVM and RF models showed the greatest sensitivity, with a value of 0.941, and the GNB model showed the lowest sensitivity (0.856). Considering that precision and sensitivity are often contradictory, we calculated the *F* score, an evaluation indicator that weighed precision and sensitivity. The top 3 *F* score models were RF (0.947), SVM (0.935), and LR (0.925). The AUC illustrated that SVM and RF had the best predictive performance, with AUC values of 0.949 (95% CI 0.911-0.953) and 0.938 (95% CI 0.914-0.947), respectively. The other models are 0.900 above. The details are shown in Figure 2. Overall, the best-performing model was RF, followed by SVM, LR, and XGBoost. Further, the DCA curves (Figure 2) demonstrate that the RF and SVM models exhibited a greater net benefit along with the threshold probability compared with other models. The accuracy of 9 independent ML models and the stacking model ranked first and second using 10-fold cross-validation are shown in Table S3 in Multimedia Appendix 1. The lack of large discrepancies in each fold validation displayed the good stability of the prediction models.

**Table 1:** The predictive performance of the 9 independent models.

Model	Features	AUC^a^ (95% CI)	Accuracy	Precision	Recall	*F* score
GNB^b^	9	0.921 (0.916-0.927)	0.859	0.949	0.856	0.900
KNN^c^	15	0.905 (0.830-0.923)	0.884	0.922	0.922	0.922
DT^d^	15	0.905 (0.822-0.922)	0.859	0.903	0.909	0.906
LR^e^	15	0.942 (0.923-0.948)	0.888	0.928	0.922	0.925
SVM^f^	15	0.949 (0.911-0.953)	0.903	0.929	0.941	0.935
RF^g^	15	0.938 (0.914-0.947)	0.922	0.953	0.941	0.947
XGBoost^h^	15	0.941 (0.904-0.945)	0.884	0.911	0.935	0.922
Lightgbm^i^	15	0.940 (0.912-0.945)	0.884	0.923	0.915	0.921
MLP^j^	15	0.951 (0.928-0.957)	0.884	0.939	0.902	0.920

^a^AUC: area under the receiver operating characteristic curve.

^b^GNB: Gaussian Naïve Bayesian.

^c^KNN: k-nearest neighbor.

^d^DT: decision tree.

^e^LR: logistic regression.

^f^SVM: support vector machine.

^g^RF: random forest.

^h^XGBoost: extreme gradient boosting.

^i^Lightgbm: light gradient boosting machine.

^j^MLP: multilayer perceptron.

### Feature Importance

**Figure 3:**
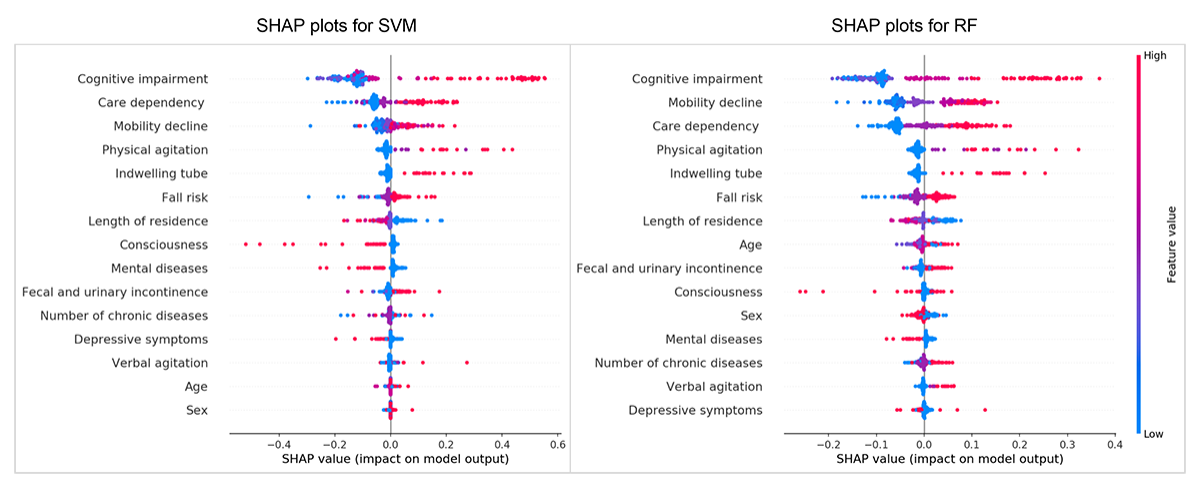
Feature importance in the SVM and RF. The SHAP value reflected the impact of features in each sample and performed their positive or negative effects. Contributing factors were ranked in descending order of importance in these plots. Each dot presented a sample; red dots presented a higher feature value, and the right side of the vertical line (ie, feature-specific SHAP values of >0) presented a higher chance of PR use. RF: random forest; SHAP: Shapley Additive Explanations; SVM: support vector machine.

Figure 3 shows the SHAP plots for SVM and RF. The results reveal that cognitive impairment, care dependency, mobility decline, physical agitation, and an indwelling tube were the strongest predictors. The SHAP plots show that lower levels of these top 5 predictors (ie, blue dots) were associated with a lower probability of PR (ie, SHAP value<0).

**Figure 4:**
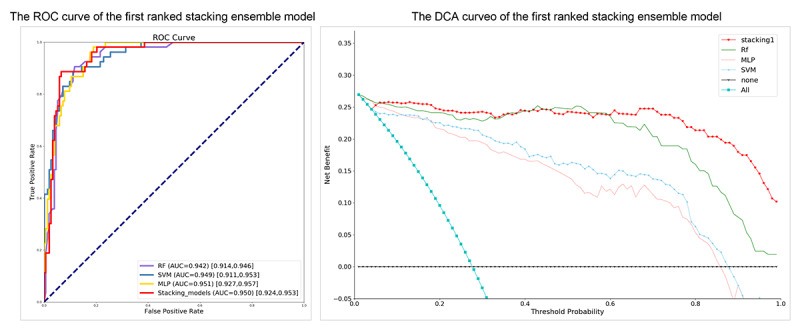
The ROC and DCA curve performance of top 1 stacking ensemble model. AUC: area under the receiver operating characteristic curve; DCA: decision curve analysis; DT: decision tree; MLP: multilayer perceptron; RF: random forest; ROC: receiver operating characteristic; SVM: support vector machine.

Stacking ensemble models were subsequently developed. A total of 510 combinations of different models were output and sorted by CEI, as shown in Multimedia Appendix 2. The stacking ensemble models that ranked first to fourth based on the CEI achieved similar performances in terms of accuracy (0.918), precision (0.942), recall (0.948), and *F* score (0.945). Overall, the first-ranked stacking ensemble model, comprising RF, SVM, and MLP, proved the best under consideration with an AUC value of 0.950 (95% CI 0.924-0.953), which is marginally higher than that of the second-ranked model (AUC 0.949, 95% CI 0.925-0.954; Figure 4). Meanwhile, the stacking model displayed a greater net benefit along with the threshold probability compared with other independent ML models (Figure 4).

## Discussion

### Principal Findings

To the best of our knowledge, this is the first study based on the systematic framework of ML techniques to generate multiple models, assess performance, and select the highest-performing models for predicting older adults’ possibility of PR use. This study demonstrates that RF and SVM displayed better performance and clinical utility than other independent ML models and that cognitive impairment, care dependency, mobility decline, physical agitation, and an indwelling tube were important contributing factors to model performance. Additionally, the model combining RF, SVM, and MLP was identified as the best model in stacking ensemble learning, which improved the stability, clinical utility, and generalization of the prediction model. These findings contribute to the early identification of older adults at high risk of PR use and targeted clinical care through timely interventions.

Among the independent ML models, SVM and RF were the 2 best-performing models, as identified by the AUC and *F* scores. In small sample data sets, like in this study, SVM may be a good choice for modeling because it can effectively handle high-dimensional spatial data. Moreover, SVM is clearer and more powerful than LR and neural networks when learning complex nonlinear data. The tree-based ML algorithms (eg, RF) are possibly more effective than neural network algorithms in terms of tabular data analysis [45]. This is similar to another study on the PR prediction model in psychiatric patients, in which RF performed better than other algorithms (ie, MLP, LR, and LASSO) [34]. Moreover, Magnowski et al [33] used fast-and-frugal tree modeling to analyze the need for restraint and seclusion; the sensitivity and specificity were only 73% and 76%, respectively. Their model’s performance was significantly lower than our model’s. The excellent performance of our ML models is primarily due to (1) an iterative grid search procedure for parameter tuning [46], (2) an iterative method for feature selection, and (3) high-quality data with many discrete and without any missing values. Although these models exhibited good performance and the best one was selected, the independent ML model was inferior to the ensemble learning model in terms of stability and generalization capacity. This limitation was overcome using our stacking-based ensemble learning algorithm [42,47]. Compared with RF, the overall predictive performance (ie, AUC) of the best stacking model combining RF, SVM, and MLP was slightly higher. The levels of sensitivity and precision of the best model are helpful in clinical practice. Additionally, the AHP-stacking algorithm helped to screen optimal models that met the needs of clinical prediction by comprehensively weighing different performance indicators [43].

In a clinical environment, the balance between predictive performance and interpretability must be considered when applying a model. The interpretation of the feature contributions is vital for ML models. Taking the 2 best independent models as examples, we carried out a feature importance analysis and used SHAP plots to visually display how features affected the prediction model. We find that cognitive impairment, mobility decline, and care dependency are the top 3 strongest predictors of PR risk. These features selected in the ML model are consistent with the clinical findings of previous studies [4,5,48]. Conversely, studies have shown that older adults who are restrained have an increased risk of cognitive decline and a decline in ability in activities of daily living [49,50]. These factors interact and result in a vicious cycle. In this study, physical agitation was one of the predictors of PR risk; however, Hofmann et al [51] report no relation between physical agitation and PR risk. Consistent with the clinical situation of using PR to prevent extubation, an indwelling tube increases the risk of PR [6]. This visual interpretation will help highlight important variables for risk prediction and preemptive and early identification of key factors. Subsequently, it will allow nursing staff to develop evidence-based interventions (eg, alternatives to PR) more timely and more targeted and thereby alleviate the risks of the first PR episode. Older adults will experience slower cognitive and ability decline by avoiding PR and engaging in persistent cognitive or ability training, the effect of which is likely to form a virtuous circle and thus reduce the risk of PR in the future as well. This is an advantage that previous studies on determining risk factors could not achieve. Additionally, as noted in the introduction, little is known about how the interplay of identified risk factors, prediction, and evaluation of PR depends on clinical experience and the decision-makers’ subjective judgment without effective and convenient risk assessment tools, which may result in the abuse of PR [52]. These prediction algorithms can automatically calculate the risk for PR without any additional workload. The PR prediction model is intended to be used as a screening tool for predicting potential PR events. An automated PR early warning system, developed based on our results, will offer clinical decision support, which deserves further study in clinical practice. The DCA curves of our models also supported these models’ potential clinical utility. Notably, PR prediction is merely a decision-support tool and cannot be relied on for conclusive results. The actual practice should follow the principles of minimized PR and prioritize alternative measures. Previous PR prediction studies of psychiatric inpatients using RF on electronic health data report limitations in adapting other electronic health record systems [34]. In this study, multiple prediction models were developed based on the real risk factors easily collected, which could be helpful in the early triage of older adults and increase the availability of data for the clinical application of the model. Overall, we believe that this work has potential impacts on risk screening, clinical decision-making, and early intervention.

This study had several strengths. First, we adopted a series of widely applied ML algorithms as well as model evaluation techniques that are lacking in existing ML for clinical prediction models. Second, we tuned the hyperparameter values for each ML algorithm identified through an iterative grid search procedure. It has been verified that hyperparameter tuning might improve the performance of models [46]. Most significantly, we presented a stacking framework and implemented it as an ensemble learning algorithm to improve the accuracy and generalization capability of the models [53]. An exhaustive method was used to form 510 combinations to select an appropriate classifier for stacking model construction. For the performance evaluation indicators of the stacking model, the base classifier selection for the stacking algorithm, based on AHP, was adopted in this study. Weight calculations based on task requirements increase the screening of models that meet clinical demands and practicability.

### Limitations

This study has several limitations. Although these models showed excellent performance in internal validation, further external validation using independent database sets is desirable. We collected non-big data from 1026 participants from 6 LTC facilities in 1 city (ie, Chongqing). Clinical large-scale data from various regions of the country should be screened to build a PR prediction model that could be applied widely. Additionally, stacking-based ensemble models were developed without feature screening; however, this had less impact on the performance of the model, as demonstrated by the high-performance results. In the future, the algorithm could be optimized to achieve feature fusion and screening. Furthermore, PR prediction would not depend only on a predictive model, regardless of the significance of the analysis performance. The pragmatic tests of these models in the real world are worth considering. This is an important challenge for all prediction models. Nevertheless, the prediction model of this study has good potential clinical utility in terms of screening assessment tools, clinical decision support, and early intervention, as mentioned in the discussion. User-centered clinical decision support systems or web-based applications based on these models remain a path to better access and improved ease of use [54].

### Conclusions

Given the decline in cognitive and daily living functions in older adults, an increasing incidence of PR and adverse effects could be expected. This may impact the quality of care and well-being of older adults. The findings of this study indicate that high-performance ML models for PR risk detection are recommended and have the potential for clinical practice. We identified the high performance and strength of the stacking ensemble learning model in predicting PR use. ML models might facilitate more effective assessments of PR risk and targeted interventions in high-risk individuals. In the future, external validation of multicenter data and the development of a web-based application for better clinical access and ease of use would be worth exploring.

## Appendix

Multimedia Appendix 1 Descriptions of predictors, risk factors related to physical restraint use identified by univariate analysis, the flow diagram of samples' selection, and accuracy of prediction models using 10-fold cross-validation test.

Multimedia Appendix 2 The predictive performance of the 510 combinations of different models.

## Abbreviations

AHP: analytic hierarchy process
AUC: area under the receiver operating characteristic curve
CEI: comprehensive evaluation indicator
DCA: decision curve analysis
DT: decision tree
GNB: Gaussian Naïve Bayesian
KNN: k-nearest neighbor
LR: logistic regression
LTC: long-term care
ML: machine learning
MLP: multilayer perceptron
PR: physical restraint
RF: random forest
SHAP: Shapley Additive Explanations
SVM: support vector machine
XGBoost: extreme gradient boosting

## Data Availability

Data can be obtained by contacting the corresponding author (MX).
