# Supplementary material for: A Risk Prediction Model for Physical Restraints Among Older Chinese Adults in Long-term Care Facilities: Machine Learning Study
Source: J Med Internet Res. 2023 Apr 6;25:e43815. doi: 10.2196/43815 (PMC10131772; doi:10.2196/43815)
Supplement: Multimedia Appendix 1 [file jmir_v25i1e43815_app1.doc]

**Multimedia Appendix 1**

**Table S1.** Descriptions of predictors.

| Variables | Description |
| --- | --- |
| Sex | Sex is divided into men and women. |
| Age | Age is divided into four subgroups, including ages ≤70 years old, 71~80 years old, 81~90 years old, and ≥91 years old. |
| Length of residence | The time older adults stay in long-term care facilities. Length of residence is divided into four subgroups, including ≤1 year, 1.1～3 years, 3.1～5 years, and ＞5 years |
| Number of chronic diseases | The total number of chronic diseases of each older adult. Three subgroups (i.e., ＜3, 3～5, ≥5) were classified. |
| Mental diseases | Mental disease is divided into three subgroups, that is, no mental disease, dementia, and others (e.g., schizophrenia, bipolar disorder, etc.). Older adults were classified as dementia if they were recorded as having any type of dementia in medical records by doctors, such as Alzheimer's disease, vascular dementia, etc. |
| Consciousness | Older adults are classified as having intact consciousness if they are sober and alert to their surroundings. Drowsiness is characterized by excessive prolongation of sleep state. with the ability to wake up and talk correctly and carry out instructions when stimulated by the outside world, and to fall back to sleep after stopping. Lethargy performance that the general external stimulus can't wake up, but the stronger stimulus can wake up briefly, and then go to sleep soon after the stimulus is weakened. Coma is a state of unarousable unconsciousness and no response to stimuli. |
| Cognitive impairment | Cognitive impairment is evaluated by the Mini-Mental State Examination (MMSE) [1]. MMSE assesses five aspects, including orientation, memory, attention and calculation, recall, and language. It is classified as intact (≥27 points), mild (21–26 points), moderate (10–20 points), or severe (0–9 points). |
| Care dependency | Care dependency is measured by the Barthel Index. The Barthel Index contains 10 items, including eating, bathing, grooming, dressing, stool control, urination control, toileting, bed and chair transfer, walking on the ground and going up and down stairs [2]. The total score is 100 points, including no dependence (100 points), mild (61–99 points), moderate (41–60 points), and severe dependence (≤40 points). |
| Mobility | Mobility is assessed by the ‘activity’ and ‘mobility’ items in the Braden Scale [3]. It is divided into three levels based on the total score, including almost no mobility (≤4 points), restricted mobility (4–7 points), and high mobility (8 points). The high her score, the better the mobility function. |
| Physical agitation | The measurement of physical agitation is to observe the frequency of its occurrence. It is divided into three levels, including no physical agitation, a few times per month, or a few times a week. |
| Verbal agitation | The measurement of verbal agitation is to observe the frequency of its occurrence. It is divided into three levels, including no verbal agitation, a few times a week, or a few times per day. |
| Depression symptoms | The Geriatric Depression Scale (GDS) is used to assess depression symptoms [4]. The GDS includes 30 “yes/no” items, with a total score of 30 points. It is classified as intact (0-10 points), mild (11-20 points), and moderate to severe (21-30 points). |
| Fecal and urinary | Older adults who have complete control of their bladder and had no indwelling urinary catheter were classified as normal. Older adults are considered to have urinary incontinence, if they are listed as being usually incontinent/occasionally incontinent, frequently incontinent or always incontinent, or had an indwelling urinary catheter. A similar classification of fecal incontinence is also used. These are extracted from medical records of older adults |
| Fall risk | Fall risk is assessed using the Morse scale [5]. The Morse scale consists of five aspects, including the history of falling, presence of secondary diagnosis, use of walking aids (cane, wheelchair, or walking frame), administration of intravenous therapy, type of gait (normal, weak, or impaired), and mental status. It is categorized as low risk (<25 points), moderate risk (25–45 points), and high risk (>45 points). |
| Indwelling tube | Older adults are classified as indwelling tubes if they are listed as having any kind of tubes in them, such as nasogastric tubes, urinary tubes, venous catheters, etc. |

**Reference:**

1. Folstein MF, Folstein SE, McHugh PR. "Mini-mental state". A practical method for grading the cognitive state of patients for the clinician. J Psychiatr Res. 1975 Nov;12(3):189-98. PMID: 1202204. doi: 10.1016/0022-3956(75)90026-6.

2. Liang Y, Welmer AK, Wang R, Song A, Fratiglioni L, Qiu C. Trends in Incidence of Disability in Activities of Daily Living in Chinese Older Adults: 1993-2006. J Am Geriatr Soc. 2017 Feb;65(2):306-12. PMID: 27682324. doi: 10.1111/jgs.14468.

3. Bergstrom N, Braden BJ, Laguzza A, Holman V. The Braden Scale for Predicting Pressure Sore Risk. Nurs Res. 1987 Jul-Aug;36(4):205-10. PMID: 3299278.

4. Chan AC. Clinical validation of the Geriatric Depression Scale (GDS): Chinese version. J Aging Health. 1996 May;8(2):238-53. PMID: 10160560. doi: 10.1177/089826439600800205.

5. Morse JM, Black C, Oberle K, Donahue P. A prospective study to identify the fall-prone patient. Soc Sci Med. 1989;28(1):81-6. PMID: 2928815. doi: 10.1016/0277-9536(89)90309-2.

**Figure S1.** Flow diagram of the participants’ selection.

A total of 1124 older adults in six LTC facilities in Chongqing, China

98 were ineligible and excluded:

Absence in the LTC facilities on the days of data collection (n=79)

Do not allowed to observe because of medical conditions (n=19)

1026 older adults eligible for the study

Older adults without PR

(n=761)

Restrained older adults

(n=265)

**Table S2.** Characteristics of samples and risk factors related to physical restraint use identified by univariate analysis (n=1026).

| **Variables** | **Total（n,%）** | **N-PR（n,%）** | **PR（n,%）** | **χ^2^** | ***p*-value** |
| --- | --- | --- | --- | --- | --- |
| **Sociodemographic characteristics** |  |  |  |  |  |
| Sex |  |  |  | 2.817 | 0.093 |
| Men | 440 (42.88) | 338 (44.42) | 102 (38.49) |  |  |
| Women | 586 (57.12) | 423 (55.58) | 163 (61.51) |  |  |
| Age (years) |  |  |  | 27.161 | ＜0.001 |
| ≤70 | 76 (7.41) | 58 (7.62) | 18 (6.79) |  |  |
| 71～80 | 249 (24.27) | 205 (26.94) | 44 (16.60) |  |  |
| 81～90 | 538 (52.44) | 401 (52.69) | 137 (51.70) |  |  |
| ≥91 | 163 (15.89) | 97 (12.75) | 66 (24.91) |  |  |
| Length of residence |  |  |  | 15.072 | 0.002 |
| ≤1 year | 266 (25.93) | 180 (23.65) | 86 (32.45) |  |  |
| 1.1～3 years | 467 (45.52) | 359 (47.17) | 108 (40.75) |  |  |
| 3.1～5 years | 221 (21.54) | 176 (23.13) | 45 (16.98) |  |  |
| ＞5 years | 72 (7.02) | 46 (6.04) | 26 (9.81) |  |  |
| **Clinical factors** |  |  |  |  |  |
| Number of chronic diseases |  |  |  | 8.18 | 0.017 |
| ＜3 | 289 (28.17) | 218 (28.65) | 71 (26.79) |  |  |
| 3～5 | 610 (59.45) | 462 (60.71) | 148 (55.85) |  |  |
| ≥5 | 127 (12.38) | 81 (10.64) | 46 (17.36) |  |  |
| Mental diseases |  |  |  | 4.788 | 0.091 |
| none | 884 (86.16) | 655 (86.07) | 229 (86.42) |  |  |
| Dementia | 117 (11.40) | 83 (10.91) | 34 (12.83) |  |  |
| others | 25 (2.44) | 23 (3.02) | 2 (0.75) |  |  |
| Consciousness |  |  |  | 65.945 | ＜0.001 |
| Intact | 923 (89.96) | 715 (93.96) | 208 (78.49) |  |  |
| Drowsiness | 87 (8.48) | 35 (4.60) | 52 (19.62) |  |  |
| Lethargy | 8 (0.78) | 3 (0.39) | 5 (1.89) |  |  |
| Coma | 8 (0.78) | 8 (1.05) | 0 (0.00) |  |  |
| Cognitive impairment |  |  |  | 405.256 | ＜0.001 |
| Intact | 387 (37.72) | 370 (48.62) | 17 (6.42) |  |  |
| Mild | 206 (20.08) | 196 (25.76) | 10 (3.77) |  |  |
| Moderate | 198 (19.30) | 128 (16.82) | 70 (26.42) |  |  |
| Severe | 235 (22.90) | 67 (8.80) | 168 (63.40) |  |  |
| Care dependency (points) |  |  |  | 333.834 | ＜0.001 |
| 61～100 | 484 (47.17) | 469 (61.63) | 15 (5.66) |  |  |
| 41～60 | 250 (24.37) | 182 (23.92) | 68 (25.66) |  |  |
| ≤40 | 292 (28.46) | 110 (14.45) | 182 (68.68) |  |  |
| Mobility (points) |  |  |  | 282.047 | ＜0.001 |
| 8 | 238 (23.20) | 235 (30.88) | 3 (1.13) |  |  |
| 7 | 221 (21.54) | 206 (27.07) | 15 (5.66) |  |  |
| 6 | 181 (17.64) | 139 (18.27) | 42 (15.85) |  |  |
| 5 | 123 (11.99) | 73 (9.59) | 50 (18.87) |  |  |
| 4 | 121 (11.79) | 50 (6.57) | 71 (26.79) |  |  |
| ＜4 | 142 (13.84) | 58 (7.62) | 84 (31.70) |  |  |
| Physical agitation |  |  |  | 102.491 | ＜0.001 |
| no | 916 (89.28) | 722 (94.88) | 194 (73.21) |  |  |
| a few times per month | 49 (4.78) | 23 (3.02) | 26 (9.81) |  |  |
| a few times a week | 61 (5.95) | 16 (2.10) | 45 (16.98) |  |  |
| Verbal agitation |  |  |  | 50.074 | ＜0.001 |
| no | 913 (88.99) | 704 (92.51) | 209 (78.87) |  |  |
| a few times a week | 65 (6.34) | 41 (5.39) | 24 (9.06) |  |  |
| a few times per day | 48 (4.68) | 16 (2.10) | 32 (12.08) |  |  |
| Depression symptoms |  |  |  | 6.582 | 0.037 |
| Intact | 860 (83.82) | 650 (85.41) | 210 (79.25) |  |  |
| Mild | 142 (13.84) | 97 (12.75) | 45 (16.98) |  |  |
| Moderate to severe | 24 (2.34) | 14 (1.84) | 10 (3.77) |  |  |
| Fecal and urinary |  |  |  | 159.226 | ＜0.001 |
| no | 705 (68.71) | 592 (77.79) | 113 (42.64) |  |  |
| fecal incontinence | 25 (2.44) | 12 (1.58) | 13 (4.91) |  |  |
| urinary incontinence | 84 (8.19) | 52 (6.83) | 32 (12.08) |  |  |
| fecal and urinary incontinence | 185 (18.03) | 78 (10.25) | 107 (40.38) |  |  |
| constipation | 27 (2.63) | 27 (3.55) | 0 (0.00) |  |  |
| Fall risk |  |  |  | 30.953 | ＜0.001 |
| Low risk | 77 (7.50) | 66 (8.67) | 11 (4.15) |  |  |
| Moderate risk | 485 (47.27) | 389 (51.12) | 96 (36.23) |  |  |
| High risk | 464 (45.22) | 306 (40.21) | 158 (59.62) |  |  |
| Indwelling tube |  |  |  | 102.439 | ＜0.001 |
| no | 939 (91.52) | 736 (96.71) | 203 (76.60) |  |  |
| yes | 87 (8.48) | 25 (3.29) | 62 (23.40) |  |  |

Notes: Abbreviations: N-PR, No Physical Restraint; PR, Physical Restraint

**Table S3**. Accuracy of prediction models using ten-fold cross-validation test.

| Accuracy | GB | KNN | DT | LR | SVM | RF | XGBoost | lightGBM | MLP | Stacking1 | Stacking2 |
| --- | --- | --- | --- | --- | --- | --- | --- | --- | --- | --- | --- |
| 1-Fold | 0.829 | 0.854 | 0.915 | 0.890 | 0.866 | 0.902 | 0.866 | 0.866 | 0.890 | 0.902 | 0.902 |
| 2-Fold | 0.890 | 0.915 | 0.878 | 0.902 | 0.902 | 0.951 | 0.915 | 0.902 | 0.902 | 0.939 | 0.927 |
| 3-Fold | 0.817 | 0.829 | 0.841 | 0.854 | 0.829 | 0.854 | 0.854 | 0.841 | 0.854 | 0.878 | 0.890 |
| 4-Fold | 0.817 | 0.841 | 0.829 | 0.841 | 0.817 | 0.854 | 0.817 | 0.793 | 0.841 | 0.854 | 0.854 |
| 5-Fold | 0.890 | 0.841 | 0.878 | 0.927 | 0.927 | 0.890 | 0.939 | 0.939 | 0.915 | 0.902 | 0.915 |
| 6-Fold | 0.841 | 0.854 | 0.878 | 0.854 | 0.866 | 0.878 | 0.866 | 0.854 | 0.854 | 0.878 | 0.890 |
| 7-Fold | 0.756 | 0.817 | 0.841 | 0.866 | 0.841 | 0.878 | 0.841 | 0.841 | 0.866 | 0.878 | 0.890 |
| 8-Fold | 0.805 | 0.805 | 0.829 | 0.829 | 0.805 | 0.866 | 0.817 | 0.829 | 0.829 | 0.854 | 0.878 |
| 9-Fold | 0.805 | 0.878 | 0.841 | 0.878 | 0.829 | 0.854 | 0.878 | 0.854 | 0.854 | 0.841 | 0.854 |
| 10-Fold | 0.768 | 0.866 | 0.841 | 0.841 | 0.854 | 0.866 | 0.878 | 0.841 | 0.854 | 0.841 | 0.829 |

Notes: Gaussian Naïve Bayesian=GNB, K-Nearest Neighbor=KNN, Decision Tree=DT, Logistic Regression=LR, Support Vector Machine= SVM, Random Forest=RF, Multilayer perceptron=MLP
