# Supplementary material for: A Risk Prediction Model for Physical Restraints Among Older Chinese Adults in Long-term Care Facilities: Machine Learning Study
Source: J Med Internet Res. 2023 Apr 6;25:e43815. doi: 10.2196/43815 (PMC10131772; doi:10.2196/43815)
Supplement: Multimedia Appendix 2 [file jmir_v25i1e43815_app2.pdf]

Model combination	Accuracy	Precision	Recall	F1-score	Cohen's Kappa	Comprehensive evaluation indicator	Weigh 1	Weigh 2	Weigh 3	Weigh 4	Order
['RF', 'SVM', 'MLP']	0.9175	0.9416	0.9477	0.9446	0.7827	0.94331014	0.061	0.2934	0.1821	0.4635	1
['DT', 'RF', 'XGBoost', 'lightGBM', 'MLP']	0.9175	0.9416	0.9477	0.9446	0.7827	0.94331014	0.061	0.2934	0.1821	0.4635	2
['DT', 'RF', 'LR', 'GNB', 'lightGBM', 'MLP']	0.9175	0.9416	0.9477	0.9446	0.7827	0.94331014	0.061	0.2934	0.1821	0.4635	3
['KNN', 'DT', 'RF', 'LR', 'XGBoost', 'lightGBM', 'MLP']	0.9175	0.9416	0.9477	0.9446	0.7827	0.94331014	0.061	0.2934	0.1821	0.4635	4
['KNN', 'DT', 'RF', 'SVM']	0.9126	0.9355	0.9477	0.9416	0.7685	0.94050993	0.061	0.2934	0.1821	0.4635	5
['KNN', 'DT', 'RF', 'XGBoost', 'MLP']	0.9126	0.9355	0.9477	0.9416	0.7685	0.94050993	0.061	0.2934	0.1821	0.4635	6
['KNN', 'RF', 'SVM', 'GNB', 'XGBoost']	0.9126	0.9355	0.9477	0.9416	0.7685	0.94050993	0.061	0.2934	0.1821	0.4635	7
['KNN', 'LR', 'XGBoost', 'lightGBM', 'MLP']	0.9126	0.9355	0.9477	0.9416	0.7685	0.94050993	0.061	0.2934	0.1821	0.4635	8
['KNN', 'DT', 'RF', 'SVM', 'GNB', 'MLP']	0.9126	0.9355	0.9477	0.9416	0.7685	0.94050993	0.061	0.2934	0.1821	0.4635	9
['KNN', 'DT', 'RF', 'GNB', 'lightGBM', 'MLP']	0.9126	0.9355	0.9477	0.9416	0.7685	0.94050993	0.061	0.2934	0.1821	0.4635	10
['KNN', 'SVM', 'LR', 'XGBoost', 'lightGBM', 'MLP']	0.9126	0.9355	0.9477	0.9416	0.7685	0.94050993	0.061	0.2934	0.1821	0.4635	11
['KNN', 'RF', 'MLP']	0.9126	0.9412	0.9412	0.9412	0.7714	0.9394554	0.061	0.2934	0.1821	0.4635	12
['KNN', 'RF', 'SVM', 'lightGBM']	0.9126	0.9412	0.9412	0.9412	0.7714	0.9394554	0.061	0.2934	0.1821	0.4635	13
['DT', 'RF', 'SVM', 'MLP']	0.9126	0.9412	0.9412	0.9412	0.7714	0.9394554	0.061	0.2934	0.1821	0.4635	14
['DT', 'RF', 'LR', 'XGBoost']	0.9126	0.9412	0.9412	0.9412	0.7714	0.9394554	0.061	0.2934	0.1821	0.4635	15
['DT', 'RF', 'XGBoost', 'lightGBM']	0.9126	0.9412	0.9412	0.9412	0.7714	0.9394554	0.061	0.2934	0.1821	0.4635	16
['DT', 'RF', 'LR', 'GNB', 'MLP']	0.9126	0.9412	0.9412	0.9412	0.7714	0.9394554	0.061	0.2934	0.1821	0.4635	17
['KNN', 'DT', 'RF', 'LR', 'XGBoost', 'MLP']	0.9126	0.9412	0.9412	0.9412	0.7714	0.9394554	0.061	0.2934	0.1821	0.4635	18
['KNN', 'DT', 'RF', 'XGBoost', 'lightGBM', 'MLP']	0.9126	0.9412	0.9412	0.9412	0.7714	0.9394554	0.061	0.2934	0.1821	0.4635	19
['KNN', 'RF', 'SVM', 'GNB', 'XGBoost', 'lightGBM']	0.9126	0.9412	0.9412	0.9412	0.7714	0.9394554	0.061	0.2934	0.1821	0.4635	20
['KNN', 'DT', 'RF', 'SVM', 'LR', 'GNB', 'XGBoost']	0.9126	0.9412	0.9412	0.9412	0.7714	0.9394554	0.061	0.2934	0.1821	0.4635	21
['KNN', 'DT', 'RF', 'LR', 'GNB', 'XGBoost', 'lightGBM']	0.9126	0.9412	0.9412	0.9412	0.7714	0.9394554	0.061	0.2934	0.1821	0.4635	22
['KNN', 'DT', 'RF', 'SVM', 'GNB', 'XGBoost', 'lightGBM', 'MLP']	0.9126	0.9412	0.9412	0.9412	0.7714	0.9394554	0.061	0.2934	0.1821	0.4635	23
['KNN', 'DT', 'RF', 'LR', 'GNB', 'XGBoost', 'lightGBM', 'MLP']	0.9126	0.9412	0.9412	0.9412	0.7714	0.9394554	0.061	0.2934	0.1821	0.4635	24
['RF']	0.9078	0.9241	0.9542	0.9389	0.751	0.93879684	0.061	0.2934	0.1821	0.4635	25
['RF', 'SVM']	0.9078	0.9295	0.9477	0.9385	0.7541	0.93768768	0.061	0.2934	0.1821	0.4635	26
['KNN', 'lightGBM', 'MLP']	0.9078	0.9295	0.9477	0.9385	0.7541	0.93768768	0.061	0.2934	0.1821	0.4635	27
['KNN', 'DT', 'SVM', 'MLP']	0.9078	0.9295	0.9477	0.9385	0.7541	0.93768768	0.061	0.2934	0.1821	0.4635	28
['KNN', 'RF', 'XGBoost', 'MLP']	0.9078	0.9295	0.9477	0.9385	0.7541	0.93768768	0.061	0.2934	0.1821	0.4635	29
['KNN', 'SVM', 'XGBoost', 'MLP']	0.9078	0.9295	0.9477	0.9385	0.7541	0.93768768	0.061	0.2934	0.1821	0.4635	30
['KNN', 'SVM', 'lightGBM', 'MLP']	0.9078	0.9295	0.9477	0.9385	0.7541	0.93768768	0.061	0.2934	0.1821	0.4635	31
['DT', 'RF', 'SVM', 'XGBoost']	0.9078	0.9295	0.9477	0.9385	0.7541	0.93768768	0.061	0.2934	0.1821	0.4635	32
['KNN', 'DT', 'LR', 'MLP']	0.9078	0.9351	0.9412	0.9381	0.7572	0.93661494	0.061	0.2934	0.1821	0.4635	33
['KNN', 'DT', 'XGBoost', 'MLP']	0.9078	0.9351	0.9412	0.9381	0.7572	0.93661494	0.061	0.2934	0.1821	0.4635	34
['KNN', 'XGBoost', 'lightGBM', 'MLP']	0.9078	0.9351	0.9412	0.9381	0.7572	0.93661494	0.061	0.2934	0.1821	0.4635	35
['DT', 'RF', 'GNB', 'lightGBM']	0.9078	0.9351	0.9412	0.9381	0.7572	0.93661494	0.061	0.2934	0.1821	0.4635	36
['DT', 'RF', 'XGBoost', 'MLP']	0.9078	0.9351	0.9412	0.9381	0.7572	0.93661494	0.061	0.2934	0.1821	0.4635	37
['RF', 'SVM', 'XGBoost', 'MLP']	0.9078	0.9351	0.9412	0.9381	0.7572	0.93661494	0.061	0.2934	0.1821	0.4635	38
['KNN', 'DT', 'RF', 'SVM', 'MLP']	0.9078	0.9351	0.9412	0.9381	0.7572	0.93661494	0.061	0.2934	0.1821	0.4635	39
['KNN', 'DT', 'RF', 'GNB', 'MLP']	0.9078	0.9351	0.9412	0.9381	0.7572	0.93661494	0.061	0.2934	0.1821	0.4635	40
['KNN', 'DT', 'SVM', 'LR', 'lightGBM']	0.9078	0.9351	0.9412	0.9381	0.7572	0.93661494	0.061	0.2934	0.1821	0.4635	41
['KNN', 'DT', 'XGBoost', 'lightGBM', 'MLP']	0.9078	0.9351	0.9412	0.9381	0.7572	0.93661494	0.061	0.2934	0.1821	0.4635	42
['KNN', 'SVM', 'LR', 'GNB', 'lightGBM']	0.9078	0.9351	0.9412	0.9381	0.7572	0.93661494	0.061	0.2934	0.1821	0.4635	43
['KNN', 'SVM', 'XGBoost', 'lightGBM', 'MLP']	0.9078	0.9351	0.9412	0.9381	0.7572	0.93661494	0.061	0.2934	0.1821	0.4635	44
['KNN', 'LR', 'GNB', 'XGBoost', 'lightGBM']	0.9078	0.9351	0.9412	0.9381	0.7572	0.93661494	0.061	0.2934	0.1821	0.4635	45
['KNN', 'LR', 'GNB', 'lightGBM', 'MLP']	0.9078	0.9351	0.9412	0.9381	0.7572	0.93661494	0.061	0.2934	0.1821	0.4635	46
['DT', 'RF', 'SVM', 'GNB', 'XGBoost']	0.9078	0.9351	0.9412	0.9381	0.7572	0.93661494	0.061	0.2934	0.1821	0.4635	47
['DT', 'RF', 'SVM', 'lightGBM', 'MLP']	0.9078	0.9351	0.9412	0.9381	0.7572	0.93661494	0.061	0.2934	0.1821	0.4635	48
['DT', 'RF', 'LR', 'XGBoost', 'MLP']	0.9078	0.9351	0.9412	0.9381	0.7572	0.93661494	0.061	0.2934	0.1821	0.4635	49
['DT', 'RF', 'GNB', 'XGBoost', 'MLP']	0.9078	0.9351	0.9412	0.9381	0.7572	0.93661494	0.061	0.2934	0.1821	0.4635	50
['KNN', 'DT', 'RF', 'SVM', 'XGBoost', 'MLP']	0.9078	0.9351	0.9412	0.9381	0.7572	0.93661494	0.061	0.2934	0.1821	0.4635	51
['KNN', 'DT', 'RF', 'SVM', 'lightGBM', 'MLP']	0.9078	0.9351	0.9412	0.9381	0.7572	0.93661494	0.061	0.2934	0.1821	0.4635	52
['KNN', 'DT', 'RF', 'LR', 'lightGBM', 'MLP']	0.9078	0.9351	0.9412	0.9381	0.7572	0.93661494	0.061	0.2934	0.1821	0.4635	53
['KNN', 'DT', 'SVM', 'LR', 'XGBoost', 'lightGBM']	0.9078	0.9351	0.9412	0.9381	0.7572	0.93661494	0.061	0.2934	0.1821	0.4635	54
['KNN', 'RF', 'SVM', 'XGBoost', 'lightGBM', 'MLP']	0.9078	0.9351	0.9412	0.9381	0.7572	0.93661494	0.061	0.2934	0.1821	0.4635	55
['KNN', 'SVM', 'LR', 'GNB', 'XGBoost', 'lightGBM']	0.9078	0.9351	0.9412	0.9381	0.7572	0.93661494	0.061	0.2934	0.1821	0.4635	56
['KNN', 'SVM', 'LR', 'GNB', 'lightGBM', 'MLP']	0.9078	0.9351	0.9412	0.9381	0.7572	0.93661494	0.061	0.2934	0.1821	0.4635	57
['DT', 'RF', 'LR', 'GNB', 'XGBoost', 'MLP']	0.9078	0.9351	0.9412	0.9381	0.7572	0.93661494	0.061	0.2934	0.1821	0.4635	58
['KNN', 'DT', 'RF', 'SVM', 'LR', 'XGBoost', 'MLP']	0.9078	0.9351	0.9412	0.9381	0.7572	0.93661494	0.061	0.2934	0.1821	0.4635	59
['KNN', 'DT', 'RF', 'SVM', 'GNB', 'lightGBM', 'MLP']	0.9078	0.9351	0.9412	0.9381	0.7572	0.93661494	0.061	0.2934	0.1821	0.4635	60
['KNN', 'DT', 'RF', 'SVM', 'XGBoost', 'lightGBM', 'MLP']	0.9078	0.9351	0.9412	0.9381	0.7572	0.93661494	0.061	0.2934	0.1821	0.4635	61
['DT', 'RF']	0.9078	0.9408	0.9346	0.9377	0.7601	0.93553107	0.061	0.2934	0.1821	0.4635	62
['RF', 'MLP']	0.9078	0.9408	0.9346	0.9377	0.7601	0.93553107	0.061	0.2934	0.1821	0.4635	63
['RF', 'GNB', 'lightGBM']	0.9078	0.9408	0.9346	0.9377	0.7601	0.93553107	0.061	0.2934	0.1821	0.4635	64
['KNN', 'DT', 'RF', 'LR']	0.9078	0.9408	0.9346	0.9377	0.7601	0.93553107	0.061	0.2934	0.1821	0.4635	65
['DT', 'RF', 'LR', 'GNB']	0.9078	0.9408	0.9346	0.9377	0.7601	0.93553107	0.061	0.2934	0.1821	0.4635	66
['DT', 'RF', 'GNB', 'MLP']	0.9078	0.9408	0.9346	0.9377	0.7601	0.93553107	0.061	0.2934	0.1821	0.4635	67
['RF', 'SVM', 'GNB', 'MLP']	0.9078	0.9408	0.9346	0.9377	0.7601	0.93553107	0.061	0.2934	0.1821	0.4635	68
['KNN', 'DT', 'RF', 'LR', 'GNB']	0.9078	0.9408	0.9346	0.9377	0.7601	0.93553107	0.061	0.2934	0.1821	0.4635	69
['KNN', 'DT', 'RF', 'lightGBM', 'MLP']	0.9078	0.9408	0.9346	0.9377	0.7601	0.93553107	0.061	0.2934	0.1821	0.4635	70
['KNN', 'RF', 'GNB', 'lightGBM', 'MLP']	0.9078	0.9408	0.9346	0.9377	0.7601	0.93553107	0.061	0.2934	0.1821	0.4635	71
['DT', 'RF', 'SVM', 'GNB', 'MLP']	0.9078	0.9408	0.9346	0.9377	0.7601	0.93553107	0.061	0.2934	0.1821	0.4635	72
['RF', 'LR', 'GNB', 'XGBoost', 'lightGBM']	0.9078	0.9408	0.9346	0.9377	0.7601	0.93553107	0.061	0.2934	0.1821	0.4635	73
['RF', 'LR', 'GNB', 'lightGBM', 'MLP']	0.9078	0.9408	0.9346	0.9377	0.7601	0.93553107	0.061	0.2934	0.1821	0.4635	74
['KNN', 'DT', 'RF', 'SVM', 'LR', 'GNB']	0.9078	0.9408	0.9346	0.9377	0.7601	0.93553107	0.061	0.2934	0.1821	0.4635	75
['KNN', 'DT', 'RF', 'LR', 'GNB', 'lightGBM']	0.9078	0.9408	0.9346	0.9377	0.7601	0.93553107	0.061	0.2934	0.1821	0.4635	76
['KNN', 'DT', 'RF', 'LR', 'XGBoost', 'lightGBM']	0.9078	0.9408	0.9346	0.9377	0.7601	0.93553107	0.061	0.2934	0.1821	0.4635	77
['KNN', 'DT', 'RF', 'GNB', 'XGBoost', 'lightGBM']	0.9078	0.9408	0.9346	0.9377	0.7601	0.93553107	0.061	0.2934	0.1821	0.4635	78
['RF', 'SVM', 'LR', 'GNB', 'lightGBM', 'MLP']	0.9078	0.9408	0.9346	0.9377	0.7601	0.93553107	0.061	0.2934	0.1821	0.4635	79
['KNN', 'DT', 'RF', 'SVM', 'GNB', 'XGBoost', 'MLP']	0.9078	0.9408	0.9346	0.9377	0.7601	0.93553107	0.061	0.2934	0.1821	0.4635	80
['DT', 'RF', 'SVM', 'LR', 'GNB', 'XGBoost', 'MLP']	0.9078	0.9408	0.9346	0.9377	0.7601	0.93553107	0.061	0.2934	0.1821	0.4635	81
['DT', 'RF', 'SVM', 'LR', 'XGBoost', 'lightGBM', 'MLP']	0.9078	0.9408	0.9346	0.9377	0.7601	0.93553107	0.061	0.2934	0.1821	0.4635	82
['KNN', 'DT', 'RF', 'SVM', 'LR', 'GNB', 'XGBoost', 'lightGBM']	0.9078	0.9408	0.9346	0.9377	0.7601	0.93553107	0.061	0.2934	0.1821	0.4635	83
['KNN', 'DT', 'RF']	0.9029	0.9236	0.9477	0.9355	0.7395	0.93492389	0.061	0.2934	0.1821	0.4635	84
['KNN', 'SVM', 'lightGBM']	0.9029	0.9236	0.9477	0.9355	0.7395	0.93492389	0.061	0.2934	0.1821	0.4635	85
['KNN', 'RF', 'SVM', 'XGBoost']	0.9029	0.9236	0.9477	0.9355	0.7395	0.93492389	0.061	0.2934	0.1821	0.4635	86
['RF', 'GNB', 'MLP']	0.9078	0.9467	0.9281	0.9373	0.763	0.93451296	0.061	0.2934	0.1821	0.4635	87

['RF', 'GNB', 'lightGBM', 'MLP']	0.9078	0.9467	0.9281	0.9373	0.763	0.93451296	0.061	0.2934	0.1821	0.4635	88
['KNN', 'RF', 'SVM', 'GNB', 'MLP']	0.9078	0.9467	0.9281	0.9373	0.763	0.93451296	0.061	0.2934	0.1821	0.4635	89
['KNN', 'RF', 'SVM', 'LR', 'GNB', 'MLP']	0.9078	0.9467	0.9281	0.9373	0.763	0.93451296	0.061	0.2934	0.1821	0.4635	90
['RF', 'SVM', 'GNB', 'XGBoost', 'lightGBM', 'MLP']	0.9078	0.9467	0.9281	0.9373	0.763	0.93451296	0.061	0.2934	0.1821	0.4635	91
['RF', 'XGBoost']	0.9029	0.929	0.9412	0.9351	0.7428	0.93381473	0.061	0.2934	0.1821	0.4635	92
['KNN', 'DT', 'MLP']	0.9029	0.929	0.9412	0.9351	0.7428	0.93381473	0.061	0.2934	0.1821	0.4635	93
['KNN', 'XGBoost', 'MLP']	0.9029	0.929	0.9412	0.9351	0.7428	0.93381473	0.061	0.2934	0.1821	0.4635	94
['DT', 'RF', 'SVM']	0.9029	0.929	0.9412	0.9351	0.7428	0.93381473	0.061	0.2934	0.1821	0.4635	95
['RF', 'SVM', 'XGBoost']	0.9029	0.929	0.9412	0.9351	0.7428	0.93381473	0.061	0.2934	0.1821	0.4635	96
['KNN', 'RF', 'SVM', 'GNB']	0.9029	0.929	0.9412	0.9351	0.7428	0.93381473	0.061	0.2934	0.1821	0.4635	97
['KNN', 'SVM', 'LR', 'XGBoost']	0.9029	0.929	0.9412	0.9351	0.7428	0.93381473	0.061	0.2934	0.1821	0.4635	98
['KNN', 'LR', 'GNB', 'lightGBM']	0.9029	0.929	0.9412	0.9351	0.7428	0.93381473	0.061	0.2934	0.1821	0.4635	99
['KNN', 'LR', 'lightGBM', 'MLP']	0.9029	0.929	0.9412	0.9351	0.7428	0.93381473	0.061	0.2934	0.1821	0.4635	100
['KNN', 'DT', 'SVM', 'LR', 'XGBoost']	0.9029	0.929	0.9412	0.9351	0.7428	0.93381473	0.061	0.2934	0.1821	0.4635	101
['KNN', 'DT', 'SVM', 'LR', 'MLP']	0.9029	0.929	0.9412	0.9351	0.7428	0.93381473	0.061	0.2934	0.1821	0.4635	102
['KNN', 'DT', 'SVM', 'XGBoost', 'MLP']	0.9029	0.929	0.9412	0.9351	0.7428	0.93381473	0.061	0.2934	0.1821	0.4635	103
['KNN', 'RF', 'SVM', 'XGBoost', 'MLP']	0.9029	0.929	0.9412	0.9351	0.7428	0.93381473	0.061	0.2934	0.1821	0.4635	104
['KNN', 'SVM', 'LR', 'lightGBM', 'MLP']	0.9029	0.929	0.9412	0.9351	0.7428	0.93381473	0.061	0.2934	0.1821	0.4635	105
['KNN', 'DT', 'SVM', 'LR', 'XGBoost', 'MLP']	0.9029	0.929	0.9412	0.9351	0.7428	0.93381473	0.061	0.2934	0.1821	0.4635	106
['RF', 'lightGBM']	0.9029	0.9346	0.9346	0.9346	0.746	0.9326663	0.061	0.2934	0.1821	0.4635	107
['DT', 'RF', 'MLP']	0.9029	0.9346	0.9346	0.9346	0.746	0.9326663	0.061	0.2934	0.1821	0.4635	108
['KNN', 'DT', 'RF', 'lightGBM']	0.9029	0.9346	0.9346	0.9346	0.746	0.9326663	0.061	0.2934	0.1821	0.4635	109
['KNN', 'DT', 'RF', 'MLP']	0.9029	0.9346	0.9346	0.9346	0.746	0.9326663	0.061	0.2934	0.1821	0.4635	110
['KNN', 'DT', 'LR', 'XGBoost']	0.9029	0.9346	0.9346	0.9346	0.746	0.9326663	0.061	0.2934	0.1821	0.4635	111
['KNN', 'DT', 'LR', 'lightGBM']	0.9029	0.9346	0.9346	0.9346	0.746	0.9326663	0.061	0.2934	0.1821	0.4635	112
['KNN', 'DT', 'lightGBM', 'MLP']	0.9029	0.9346	0.9346	0.9346	0.746	0.9326663	0.061	0.2934	0.1821	0.4635	113
['KNN', 'RF', 'SVM', 'LR']	0.9029	0.9346	0.9346	0.9346	0.746	0.9326663	0.061	0.2934	0.1821	0.4635	114
['KNN', 'RF', 'SVM', 'MLP']	0.9029	0.9346	0.9346	0.9346	0.746	0.9326663	0.061	0.2934	0.1821	0.4635	115
['KNN', 'LR', 'GNB', 'MLP']	0.9029	0.9346	0.9346	0.9346	0.746	0.9326663	0.061	0.2934	0.1821	0.4635	116
['DT', 'RF', 'SVM', 'LR']	0.9029	0.9346	0.9346	0.9346	0.746	0.9326663	0.061	0.2934	0.1821	0.4635	117
['DT', 'RF', 'LR', 'MLP']	0.9029	0.9346	0.9346	0.9346	0.746	0.9326663	0.061	0.2934	0.1821	0.4635	118
['RF', 'SVM', 'LR', 'lightGBM']	0.9029	0.9346	0.9346	0.9346	0.746	0.9326663	0.061	0.2934	0.1821	0.4635	119
['RF', 'SVM', 'GNB', 'XGBoost']	0.9029	0.9346	0.9346	0.9346	0.746	0.9326663	0.061	0.2934	0.1821	0.4635	120
['RF', 'SVM', 'lightGBM', 'MLP']	0.9029	0.9346	0.9346	0.9346	0.746	0.9326663	0.061	0.2934	0.1821	0.4635	121
['KNN', 'DT', 'RF', 'SVM', 'XGBoost']	0.9029	0.9346	0.9346	0.9346	0.746	0.9326663	0.061	0.2934	0.1821	0.4635	122
['KNN', 'DT', 'RF', 'LR', 'XGBoost']	0.9029	0.9346	0.9346	0.9346	0.746	0.9326663	0.061	0.2934	0.1821	0.4635	123
['KNN', 'DT', 'RF', 'LR', 'MLP']	0.9029	0.9346	0.9346	0.9346	0.746	0.9326663	0.061	0.2934	0.1821	0.4635	124
['KNN', 'DT', 'SVM', 'lightGBM', 'MLP']	0.9029	0.9346	0.9346	0.9346	0.746	0.9326663	0.061	0.2934	0.1821	0.4635	125
['KNN', 'DT', 'LR', 'XGBoost', 'lightGBM']	0.9029	0.9346	0.9346	0.9346	0.746	0.9326663	0.061	0.2934	0.1821	0.4635	126
['KNN', 'DT', 'LR', 'lightGBM', 'MLP']	0.9029	0.9346	0.9346	0.9346	0.746	0.9326663	0.061	0.2934	0.1821	0.4635	127
['KNN', 'RF', 'SVM', 'LR', 'lightGBM']	0.9029	0.9346	0.9346	0.9346	0.746	0.9326663	0.061	0.2934	0.1821	0.4635	128
['KNN', 'RF', 'SVM', 'GNB', 'lightGBM']	0.9029	0.9346	0.9346	0.9346	0.746	0.9326663	0.061	0.2934	0.1821	0.4635	129
['KNN', 'RF', 'SVM', 'lightGBM', 'MLP']	0.9029	0.9346	0.9346	0.9346	0.746	0.9326663	0.061	0.2934	0.1821	0.4635	130
['KNN', 'RF', 'LR', 'XGBoost', 'lightGBM']	0.9029	0.9346	0.9346	0.9346	0.746	0.9326663	0.061	0.2934	0.1821	0.4635	131
['KNN', 'RF', 'GNB', 'XGBoost', 'lightGBM']	0.9029	0.9346	0.9346	0.9346	0.746	0.9326663	0.061	0.2934	0.1821	0.4635	132
['KNN', 'RF', 'GNB', 'XGBoost', 'MLP']	0.9029	0.9346	0.9346	0.9346	0.746	0.9326663	0.061	0.2934	0.1821	0.4635	133
['KNN', 'RF', 'XGBoost', 'lightGBM', 'MLP']	0.9029	0.9346	0.9346	0.9346	0.746	0.9326663	0.061	0.2934	0.1821	0.4635	134
['KNN', 'GNB', 'XGBoost', 'lightGBM', 'MLP']	0.9029	0.9346	0.9346	0.9346	0.746	0.9326663	0.061	0.2934	0.1821	0.4635	135
['DT', 'RF', 'SVM', 'LR', 'GNB']	0.9029	0.9346	0.9346	0.9346	0.746	0.9326663	0.061	0.2934	0.1821	0.4635	136
['DT', 'RF', 'SVM', 'LR', 'XGBoost']	0.9029	0.9346	0.9346	0.9346	0.746	0.9326663	0.061	0.2934	0.1821	0.4635	137
['DT', 'RF', 'SVM', 'GNB', 'lightGBM']	0.9029	0.9346	0.9346	0.9346	0.746	0.9326663	0.061	0.2934	0.1821	0.4635	138
['DT', 'RF', 'SVM', 'XGBoost', 'MLP']	0.9029	0.9346	0.9346	0.9346	0.746	0.9326663	0.061	0.2934	0.1821	0.4635	139
['DT', 'RF', 'LR', 'GNB', 'XGBoost']	0.9029	0.9346	0.9346	0.9346	0.746	0.9326663	0.061	0.2934	0.1821	0.4635	140
['RF', 'SVM', 'LR', 'GNB', 'XGBoost']	0.9029	0.9346	0.9346	0.9346	0.746	0.9326663	0.061	0.2934	0.1821	0.4635	141
['RF', 'SVM', 'GNB', 'XGBoost', 'lightGBM']	0.9029	0.9346	0.9346	0.9346	0.746	0.9326663	0.061	0.2934	0.1821	0.4635	142
['RF', 'SVM', 'GNB', 'XGBoost', 'MLP']	0.9029	0.9346	0.9346	0.9346	0.746	0.9326663	0.061	0.2934	0.1821	0.4635	143
['KNN', 'DT', 'RF', 'SVM', 'LR', 'MLP']	0.9029	0.9346	0.9346	0.9346	0.746	0.9326663	0.061	0.2934	0.1821	0.4635	144
['KNN', 'DT', 'RF', 'SVM', 'XGBoost', 'lightGBM']	0.9029	0.9346	0.9346	0.9346	0.746	0.9326663	0.061	0.2934	0.1821	0.4635	145
['KNN', 'DT', 'RF', 'LR', 'GNB', 'XGBoost']	0.9029	0.9346	0.9346	0.9346	0.746	0.9326663	0.061	0.2934	0.1821	0.4635	146
['KNN', 'DT', 'RF', 'LR', 'GNB', 'MLP']	0.9029	0.9346	0.9346	0.9346	0.746	0.9326663	0.061	0.2934	0.1821	0.4635	147
['KNN', 'DT', 'SVM', 'LR', 'lightGBM', 'MLP']	0.9029	0.9346	0.9346	0.9346	0.746	0.9326663	0.061	0.2934	0.1821	0.4635	148
['KNN', 'DT', 'SVM', 'XGBoost', 'lightGBM', 'MLP']	0.9029	0.9346	0.9346	0.9346	0.746	0.9326663	0.061	0.2934	0.1821	0.4635	149
['KNN', 'DT', 'LR', 'GNB', 'XGBoost', 'MLP']	0.9029	0.9346	0.9346	0.9346	0.746	0.9326663	0.061	0.2934	0.1821	0.4635	150
['KNN', 'RF', 'LR', 'XGBoost', 'lightGBM', 'MLP']	0.9029	0.9346	0.9346	0.9346	0.746	0.9326663	0.061	0.2934	0.1821	0.4635	151
['KNN', 'RF', 'GNB', 'XGBoost', 'lightGBM', 'MLP']	0.9029	0.9346	0.9346	0.9346	0.746	0.9326663	0.061	0.2934	0.1821	0.4635	152
['KNN', 'SVM', 'GNB', 'XGBoost', 'lightGBM', 'MLP']	0.9029	0.9346	0.9346	0.9346	0.746	0.9326663	0.061	0.2934	0.1821	0.4635	153
['KNN', 'LR', 'GNB', 'XGBoost', 'lightGBM', 'MLP']	0.9029	0.9346	0.9346	0.9346	0.746	0.9326663	0.061	0.2934	0.1821	0.4635	154
['KNN', 'DT', 'RF', 'GNB', 'XGBoost', 'lightGBM', 'MLP']	0.9029	0.9346	0.9346	0.9346	0.746	0.9326663	0.061	0.2934	0.1821	0.4635	155
['KNN', 'DT', 'SVM', 'LR', 'GNB', 'XGBoost', 'MLP']	0.9029	0.9346	0.9346	0.9346	0.746	0.9326663	0.061	0.2934	0.1821	0.4635	156
['KNN', 'SVM', 'LR', 'GNB', 'XGBoost', 'lightGBM', 'MLP']	0.9029	0.9346	0.9346	0.9346	0.746	0.9326663	0.061	0.2934	0.1821	0.4635	157
['DT', 'RF', 'SVM', 'GNB', 'XGBoost', 'lightGBM', 'MLP']	0.9029	0.9346	0.9346	0.9346	0.746	0.9326663	0.061	0.2934	0.1821	0.4635	158
['KNN', 'DT', 'RF', 'SVM', 'LR', 'GNB', 'lightGBM', 'MLP']	0.9029	0.9346	0.9346	0.9346	0.746	0.9326663	0.061	0.2934	0.1821	0.4635	159
['KNN', 'DT', 'RF', 'SVM', 'LR', 'XGBoost', 'lightGBM', 'MLP']	0.9029	0.9346	0.9346	0.9346	0.746	0.9326663	0.061	0.2934	0.1821	0.4635	160
['DT', 'RF', 'SVM', 'LR', 'GNB', 'XGBoost', 'lightGBM', 'MLP']	0.9029	0.9346	0.9346	0.9346	0.746	0.9326663	0.061	0.2934	0.1821	0.4635	161
['SVM', 'MLP']	0.8981	0.9177	0.9477	0.9325	0.7248	0.9321662	0.061	0.2934	0.1821	0.4635	162
['KNN', 'DT', 'SVM']	0.8981	0.9177	0.9477	0.9325	0.7248	0.9321662	0.061	0.2934	0.1821	0.4635	163
['KNN', 'RF', 'SVM']	0.8981	0.9177	0.9477	0.9325	0.7248	0.9321662	0.061	0.2934	0.1821	0.4635	164
['RF', 'LR', 'GNB']	0.9029	0.9404	0.9281	0.9342	0.7491	0.93162998	0.061	0.2934	0.1821	0.4635	165
['RF', 'LR', 'lightGBM']	0.9029	0.9404	0.9281	0.9342	0.7491	0.93162998	0.061	0.2934	0.1821	0.4635	166
['RF', 'LR', 'MLP']	0.9029	0.9404	0.9281	0.9342	0.7491	0.93162998	0.061	0.2934	0.1821	0.4635	167
['KNN', 'RF', 'LR', 'GNB']	0.9029	0.9404	0.9281	0.9342	0.7491	0.93162998	0.061	0.2934	0.1821	0.4635	168
['KNN', 'RF', 'GNB', 'MLP']	0.9029	0.9404	0.9281	0.9342	0.7491	0.93162998	0.061	0.2934	0.1821	0.4635	169
['DT', 'RF', 'LR', 'lightGBM']	0.9029	0.9404	0.9281	0.9342	0.7491	0.93162998	0.061	0.2934	0.1821	0.4635	170
['RF', 'SVM', 'LR', 'GNB']	0.9029	0.9404	0.9281	0.9342	0.7491	0.93162998	0.061	0.2934	0.1821	0.4635	171
['RF', 'SVM', 'LR', 'MLP']	0.9029	0.9404	0.9281	0.9342	0.7491	0.93162998	0.061	0.2934	0.1821	0.4635	172
['RF', 'LR', 'GNB', 'XGBoost']	0.9029	0.9404	0.9281	0.9342	0.7491	0.93162998	0.061	0.2934	0.1821	0.4635	173
['RF', 'LR', 'GNB', 'lightGBM']	0.9029	0.9404	0.9281	0.9342	0.7491	0.93162998	0.061	0.2934	0.1821	0.4635	174
['RF', 'LR', 'XGBoost', 'lightGBM']	0.9029	0.9404	0.9281	0.9342	0.7491	0.93162998	0.061	0.2934	0.1821	0.4635	175
['RF', 'GNB', 'XGBoost', 'lightGBM']	0.9029	0.9404	0.9281	0.9342	0.7491	0.93162998	0.061	0.2934	0.1821	0.4635	176
['KNN', 'DT', 'RF', 'SVM', 'LR']	0.9029	0.9404	0.9281	0.9342	0.7491	0.93162998	0.061	0.2934	0.1821	0.4635	177

['KNN', 'RF', 'SVM', 'LR', 'GNB']	0.9029	0.9404	0.9281	0.9342	0.7491	0.93162998	0.061	0.2934	0.1821	0.4635	178
['KNN', 'RF', 'LR', 'GNB', 'lightGBM']	0.9029	0.9404	0.9281	0.9342	0.7491	0.93162998	0.061	0.2934	0.1821	0.4635	179
['KNN', 'RF', 'LR', 'GNB', 'MLP']	0.9029	0.9404	0.9281	0.9342	0.7491	0.93162998	0.061	0.2934	0.1821	0.4635	180
['DT', 'RF', 'LR', 'GNB', 'lightGBM']	0.9029	0.9404	0.9281	0.9342	0.7491	0.93162998	0.061	0.2934	0.1821	0.4635	181
['DT', 'RF', 'LR', 'XGBoost', 'lightGBM']	0.9029	0.9404	0.9281	0.9342	0.7491	0.93162998	0.061	0.2934	0.1821	0.4635	182
['DT', 'RF', 'GNB', 'lightGBM', 'MLP']	0.9029	0.9404	0.9281	0.9342	0.7491	0.93162998	0.061	0.2934	0.1821	0.4635	183
['RF', 'SVM', 'LR', 'XGBoost', 'lightGBM']	0.9029	0.9404	0.9281	0.9342	0.7491	0.93162998	0.061	0.2934	0.1821	0.4635	184
['RF', 'SVM', 'XGBoost', 'lightGBM', 'MLP']	0.9029	0.9404	0.9281	0.9342	0.7491	0.93162998	0.061	0.2934	0.1821	0.4635	185
['RF', 'LR', 'GNB', 'XGBoost', 'MLP']	0.9029	0.9404	0.9281	0.9342	0.7491	0.93162998	0.061	0.2934	0.1821	0.4635	186
['RF', 'GNB', 'XGBoost', 'lightGBM', 'MLP']	0.9029	0.9404	0.9281	0.9342	0.7491	0.93162998	0.061	0.2934	0.1821	0.4635	187
['KNN', 'DT', 'RF', 'SVM', 'LR', 'XGBoost']	0.9029	0.9404	0.9281	0.9342	0.7491	0.93162998	0.061	0.2934	0.1821	0.4635	188
['KNN', 'RF', 'SVM', 'LR', 'XGBoost', 'lightGBM']	0.9029	0.9404	0.9281	0.9342	0.7491	0.93162998	0.061	0.2934	0.1821	0.4635	189
['KNN', 'RF', 'SVM', 'GNB', 'XGBoost', 'MLP']	0.9029	0.9404	0.9281	0.9342	0.7491	0.93162998	0.061	0.2934	0.1821	0.4635	190
['KNN', 'RF', 'LR', 'GNB', 'XGBoost', 'lightGBM']	0.9029	0.9404	0.9281	0.9342	0.7491	0.93162998	0.061	0.2934	0.1821	0.4635	191
['DT', 'RF', 'SVM', 'LR', 'GNB', 'lightGBM']	0.9029	0.9404	0.9281	0.9342	0.7491	0.93162998	0.061	0.2934	0.1821	0.4635	192
['DT', 'RF', 'SVM', 'LR', 'GNB', 'MLP']	0.9029	0.9404	0.9281	0.9342	0.7491	0.93162998	0.061	0.2934	0.1821	0.4635	193
['DT', 'RF', 'SVM', 'LR', 'XGBoost', 'lightGBM']	0.9029	0.9404	0.9281	0.9342	0.7491	0.93162998	0.061	0.2934	0.1821	0.4635	194
['DT', 'RF', 'SVM', 'LR', 'lightGBM', 'MLP']	0.9029	0.9404	0.9281	0.9342	0.7491	0.93162998	0.061	0.2934	0.1821	0.4635	195
['DT', 'RF', 'LR', 'GNB', 'XGBoost', 'lightGBM']	0.9029	0.9404	0.9281	0.9342	0.7491	0.93162998	0.061	0.2934	0.1821	0.4635	196
['DT', 'RF', 'LR', 'XGBoost', 'lightGBM', 'MLP']	0.9029	0.9404	0.9281	0.9342	0.7491	0.93162998	0.061	0.2934	0.1821	0.4635	197
['RF', 'SVM', 'LR', 'GNB', 'XGBoost', 'lightGBM']	0.9029	0.9404	0.9281	0.9342	0.7491	0.93162998	0.061	0.2934	0.1821	0.4635	198
['RF', 'SVM', 'LR', 'XGBoost', 'lightGBM', 'MLP']	0.9029	0.9404	0.9281	0.9342	0.7491	0.93162998	0.061	0.2934	0.1821	0.4635	199
['RF', 'LR', 'GNB', 'XGBoost', 'lightGBM', 'MLP']	0.9029	0.9404	0.9281	0.9342	0.7491	0.93162998	0.061	0.2934	0.1821	0.4635	200
['KNN', 'DT', 'RF', 'SVM', 'LR', 'GNB', 'lightGBM']	0.9029	0.9404	0.9281	0.9342	0.7491	0.93162998	0.061	0.2934	0.1821	0.4635	201
['KNN', 'DT', 'RF', 'SVM', 'LR', 'XGBoost', 'lightGBM']	0.9029	0.9404	0.9281	0.9342	0.7491	0.93162998	0.061	0.2934	0.1821	0.4635	202
['KNN', 'DT', 'RF', 'LR', 'GNB', 'lightGBM', 'MLP']	0.9029	0.9404	0.9281	0.9342	0.7491	0.93162998	0.061	0.2934	0.1821	0.4635	203
['KNN', 'RF', 'SVM', 'LR', 'GNB', 'XGBoost', 'lightGBM']	0.9029	0.9404	0.9281	0.9342	0.7491	0.93162998	0.061	0.2934	0.1821	0.4635	204
['KNN', 'RF', 'SVM', 'LR', 'GNB', 'lightGBM', 'MLP']	0.9029	0.9404	0.9281	0.9342	0.7491	0.93162998	0.061	0.2934	0.1821	0.4635	205
['DT', 'RF', 'SVM', 'LR', 'GNB', 'XGBoost', 'lightGBM']	0.9029	0.9404	0.9281	0.9342	0.7491	0.93162998	0.061	0.2934	0.1821	0.4635	206
['DT', 'RF', 'SVM', 'LR', 'GNB', 'lightGBM', 'MLP']	0.9029	0.9404	0.9281	0.9342	0.7491	0.93162998	0.061	0.2934	0.1821	0.4635	207
['RF', 'SVM', 'LR', 'GNB', 'XGBoost', 'lightGBM', 'MLP']	0.9029	0.9404	0.9281	0.9342	0.7491	0.93162998	0.061	0.2934	0.1821	0.4635	208
['KNN', 'DT', 'RF', 'SVM', 'LR', 'GNB', 'XGBoost', 'MLP']	0.9029	0.9404	0.9281	0.9342	0.7491	0.93162998	0.061	0.2934	0.1821	0.4635	209
['SVM', 'GNB']	0.8981	0.9231	0.9412	0.932	0.7282	0.93101069	0.061	0.2934	0.1821	0.4635	210
['KNN', 'SVM', 'LR']	0.8981	0.9231	0.9412	0.932	0.7282	0.93101069	0.061	0.2934	0.1821	0.4635	211
['KNN', 'SVM', 'MLP']	0.8981	0.9231	0.9412	0.932	0.7282	0.93101069	0.061	0.2934	0.1821	0.4635	212
['DT', 'RF', 'XGBoost']	0.8981	0.9231	0.9412	0.932	0.7282	0.93101069	0.061	0.2934	0.1821	0.4635	213
['KNN', 'DT', 'RF', 'XGBoost']	0.8981	0.9231	0.9412	0.932	0.7282	0.93101069	0.061	0.2934	0.1821	0.4635	214
['KNN', 'SVM', 'LR', 'GNB', 'XGBoost']	0.8981	0.9231	0.9412	0.932	0.7282	0.93101069	0.061	0.2934	0.1821	0.4635	215
['KNN', 'LR', 'GNB', 'XGBoost', 'MLP']	0.8981	0.9231	0.9412	0.932	0.7282	0.93101069	0.061	0.2934	0.1821	0.4635	216
['LR', 'lightGBM']	0.8981	0.9286	0.9346	0.9316	0.7316	0.9298904	0.061	0.2934	0.1821	0.4635	217
['GNB', 'XGBoost']	0.8981	0.9286	0.9346	0.9316	0.7316	0.9298904	0.061	0.2934	0.1821	0.4635	218
['KNN', 'LR', 'XGBoost']	0.8981	0.9286	0.9346	0.9316	0.7316	0.9298904	0.061	0.2934	0.1821	0.4635	219
['RF', 'XGBoost', 'MLP']	0.8981	0.9286	0.9346	0.9316	0.7316	0.9298904	0.061	0.2934	0.1821	0.4635	220
['RF', 'lightGBM', 'MLP']	0.8981	0.9286	0.9346	0.9316	0.7316	0.9298904	0.061	0.2934	0.1821	0.4635	221
['KNN', 'DT', 'SVM', 'LR']	0.8981	0.9286	0.9346	0.9316	0.7316	0.9298904	0.061	0.2934	0.1821	0.4635	222
['KNN', 'DT', 'GNB', 'MLP']	0.8981	0.9286	0.9346	0.9316	0.7316	0.9298904	0.061	0.2934	0.1821	0.4635	223
['KNN', 'RF', 'lightGBM', 'MLP']	0.8981	0.9286	0.9346	0.9316	0.7316	0.9298904	0.061	0.2934	0.1821	0.4635	224
['KNN', 'SVM', 'LR', 'lightGBM']	0.8981	0.9286	0.9346	0.9316	0.7316	0.9298904	0.061	0.2934	0.1821	0.4635	225
['KNN', 'LR', 'XGBoost', 'lightGBM']	0.8981	0.9286	0.9346	0.9316	0.7316	0.9298904	0.061	0.2934	0.1821	0.4635	226
['KNN', 'LR', 'XGBoost', 'MLP']	0.8981	0.9286	0.9346	0.9316	0.7316	0.9298904	0.061	0.2934	0.1821	0.4635	227
['KNN', 'GNB', 'XGBoost', 'MLP']	0.8981	0.9286	0.9346	0.9316	0.7316	0.9298904	0.061	0.2934	0.1821	0.4635	228
['DT', 'RF', 'SVM', 'GNB']	0.8981	0.9286	0.9346	0.9316	0.7316	0.9298904	0.061	0.2934	0.1821	0.4635	229
['DT', 'RF', 'GNB', 'XGBoost']	0.8981	0.9286	0.9346	0.9316	0.7316	0.9298904	0.061	0.2934	0.1821	0.4635	230
['RF', 'XGBoost', 'lightGBM', 'MLP']	0.8981	0.9286	0.9346	0.9316	0.7316	0.9298904	0.061	0.2934	0.1821	0.4635	231
['KNN', 'DT', 'RF', 'SVM', 'GNB']	0.8981	0.9286	0.9346	0.9316	0.7316	0.9298904	0.061	0.2934	0.1821	0.4635	232
['KNN', 'DT', 'SVM', 'LR', 'GNB']	0.8981	0.9286	0.9346	0.9316	0.7316	0.9298904	0.061	0.2934	0.1821	0.4635	233
['KNN', 'DT', 'SVM', 'GNB', 'MLP']	0.8981	0.9286	0.9346	0.9316	0.7316	0.9298904	0.061	0.2934	0.1821	0.4635	234
['KNN', 'DT', 'GNB', 'XGBoost', 'MLP']	0.8981	0.9286	0.9346	0.9316	0.7316	0.9298904	0.061	0.2934	0.1821	0.4635	235
['KNN', 'DT', 'GNB', 'lightGBM', 'MLP']	0.8981	0.9286	0.9346	0.9316	0.7316	0.9298904	0.061	0.2934	0.1821	0.4635	236
['KNN', 'SVM', 'LR', 'GNB', 'MLP']	0.8981	0.9286	0.9346	0.9316	0.7316	0.9298904	0.061	0.2934	0.1821	0.4635	237
['KNN', 'SVM', 'LR', 'XGBoost', 'lightGBM']	0.8981	0.9286	0.9346	0.9316	0.7316	0.9298904	0.061	0.2934	0.1821	0.4635	238
['KNN', 'SVM', 'LR', 'XGBoost', 'MLP']	0.8981	0.9286	0.9346	0.9316	0.7316	0.9298904	0.061	0.2934	0.1821	0.4635	239
['KNN', 'SVM', 'GNB', 'XGBoost', 'MLP']	0.8981	0.9286	0.9346	0.9316	0.7316	0.9298904	0.061	0.2934	0.1821	0.4635	240
['DT', 'RF', 'SVM', 'LR', 'MLP']	0.8981	0.9286	0.9346	0.9316	0.7316	0.9298904	0.061	0.2934	0.1821	0.4635	241
['DT', 'RF', 'SVM', 'XGBoost', 'lightGBM']	0.8981	0.9286	0.9346	0.9316	0.7316	0.9298904	0.061	0.2934	0.1821	0.4635	242
['DT', 'RF', 'LR', 'lightGBM', 'MLP']	0.8981	0.9286	0.9346	0.9316	0.7316	0.9298904	0.061	0.2934	0.1821	0.4635	243
['KNN', 'DT', 'SVM', 'GNB', 'XGBoost', 'MLP']	0.8981	0.9286	0.9346	0.9316	0.7316	0.9298904	0.061	0.2934	0.1821	0.4635	244
['KNN', 'DT', 'GNB', 'XGBoost', 'lightGBM', 'MLP']	0.8981	0.9286	0.9346	0.9316	0.7316	0.9298904	0.061	0.2934	0.1821	0.4635	245
['KNN', 'RF', 'SVM', 'LR', 'XGBoost', 'MLP']	0.8981	0.9286	0.9346	0.9316	0.7316	0.9298904	0.061	0.2934	0.1821	0.4635	246
['KNN', 'SVM', 'LR', 'GNB', 'XGBoost', 'MLP']	0.8981	0.9286	0.9346	0.9316	0.7316	0.9298904	0.061	0.2934	0.1821	0.4635	247
['DT', 'RF', 'SVM', 'XGBoost', 'lightGBM', 'MLP']	0.8981	0.9286	0.9346	0.9316	0.7316	0.9298904	0.061	0.2934	0.1821	0.4635	248
['KNN', 'DT', 'SVM', 'GNB', 'XGBoost', 'lightGBM', 'MLP']	0.8981	0.9286	0.9346	0.9316	0.7316	0.9298904	0.061	0.2934	0.1821	0.4635	249
['SVM']	0.8932	0.9119	0.9477	0.9295	0.7098	0.92942062	0.061	0.2934	0.1821	0.4635	250
['KNN', 'GNB', 'MLP']	0.8981	0.9342	0.9281	0.9311	0.7349	0.92877131	0.061	0.2934	0.1821	0.4635	251
['DT', 'RF', 'lightGBM']	0.8981	0.9342	0.9281	0.9311	0.7349	0.92877131	0.061	0.2934	0.1821	0.4635	252
['RF', 'LR', 'XGBoost']	0.8981	0.9342	0.9281	0.9311	0.7349	0.92877131	0.061	0.2934	0.1821	0.4635	253
['RF', 'GNB', 'XGBoost']	0.8981	0.9342	0.9281	0.9311	0.7349	0.92877131	0.061	0.2934	0.1821	0.4635	254
['KNN', 'RF', 'LR', 'XGBoost']	0.8981	0.9342	0.9281	0.9311	0.7349	0.92877131	0.061	0.2934	0.1821	0.4635	255
['KNN', 'GNB', 'lightGBM', 'MLP']	0.8981	0.9342	0.9281	0.9311	0.7349	0.92877131	0.061	0.2934	0.1821	0.4635	256
['DT', 'RF', 'SVM', 'lightGBM']	0.8981	0.9342	0.9281	0.9311	0.7349	0.92877131	0.061	0.2934	0.1821	0.4635	257
['RF', 'SVM', 'LR', 'XGBoost']	0.8981	0.9342	0.9281	0.9311	0.7349	0.92877131	0.061	0.2934	0.1821	0.4635	258
['RF', 'SVM', 'GNB', 'lightGBM']	0.8981	0.9342	0.9281	0.9311	0.7349	0.92877131	0.061	0.2934	0.1821	0.4635	259
['RF', 'SVM', 'XGBoost', 'lightGBM']	0.8981	0.9342	0.9281	0.9311	0.7349	0.92877131	0.061	0.2934	0.1821	0.4635	260
['RF', 'LR', 'XGBoost', 'MLP']	0.8981	0.9342	0.9281	0.9311	0.7349	0.92877131	0.061	0.2934	0.1821	0.4635	261
['KNN', 'DT', 'RF', 'LR', 'lightGBM']	0.8981	0.9342	0.9281	0.9311	0.7349	0.92877131	0.061	0.2934	0.1821	0.4635	262
['KNN', 'DT', 'RF', 'GNB', 'XGBoost']	0.8981	0.9342	0.9281	0.9311	0.7349	0.92877131	0.061	0.2934	0.1821	0.4635	263
['KNN', 'DT', 'RF', 'GNB', 'lightGBM']	0.8981	0.9342	0.9281	0.9311	0.7349	0.92877131	0.061	0.2934	0.1821	0.4635	264
['KNN', 'DT', 'LR', 'GNB', 'XGBoost']	0.8981	0.9342	0.9281	0.9311	0.7349	0.92877131	0.061	0.2934	0.1821	0.4635	265
['KNN', 'DT', 'LR', 'GNB', 'MLP']	0.8981	0.9342	0.9281	0.9311	0.7349	0.92877131	0.061	0.2934	0.1821	0.4635	266
['KNN', 'DT', 'LR', 'XGBoost', 'MLP']	0.8981	0.9342	0.9281	0.9311	0.7349	0.92877131	0.061	0.2934	0.1821	0.4635	267

['KNN', 'RF', 'LR', 'GNB', 'XGBoost']	0.8981	0.9342	0.9281	0.9311	0.7349	0.92877131	0.061	0.2934	0.1821	0.4635	268
['KNN', 'RF', 'LR', 'lightGBM', 'MLP']	0.8981	0.9342	0.9281	0.9311	0.7349	0.92877131	0.061	0.2934	0.1821	0.4635	269
['KNN', 'SVM', 'GNB', 'lightGBM', 'MLP']	0.8981	0.9342	0.9281	0.9311	0.7349	0.92877131	0.061	0.2934	0.1821	0.4635	270
['RF', 'SVM', 'LR', 'GNB', 'MLP']	0.8981	0.9342	0.9281	0.9311	0.7349	0.92877131	0.061	0.2934	0.1821	0.4635	271
['RF', 'SVM', 'LR', 'lightGBM', 'MLP']	0.8981	0.9342	0.9281	0.9311	0.7349	0.92877131	0.061	0.2934	0.1821	0.4635	272
['RF', 'SVM', 'GNB', 'lightGBM', 'MLP']	0.8981	0.9342	0.9281	0.9311	0.7349	0.92877131	0.061	0.2934	0.1821	0.4635	273
['KNN', 'DT', 'RF', 'SVM', 'LR', 'lightGBM']	0.8981	0.9342	0.9281	0.9311	0.7349	0.92877131	0.061	0.2934	0.1821	0.4635	274
['KNN', 'DT', 'RF', 'SVM', 'GNB', 'XGBoost']	0.8981	0.9342	0.9281	0.9311	0.7349	0.92877131	0.061	0.2934	0.1821	0.4635	275
['KNN', 'DT', 'RF', 'SVM', 'GNB', 'lightGBM']	0.8981	0.9342	0.9281	0.9311	0.7349	0.92877131	0.061	0.2934	0.1821	0.4635	276
['KNN', 'DT', 'LR', 'XGBoost', 'lightGBM', 'MLP']	0.8981	0.9342	0.9281	0.9311	0.7349	0.92877131	0.061	0.2934	0.1821	0.4635	277
['KNN', 'RF', 'SVM', 'LR', 'GNB', 'lightGBM']	0.8981	0.9342	0.9281	0.9311	0.7349	0.92877131	0.061	0.2934	0.1821	0.4635	278
['KNN', 'RF', 'SVM', 'LR', 'lightGBM', 'MLP']	0.8981	0.9342	0.9281	0.9311	0.7349	0.92877131	0.061	0.2934	0.1821	0.4635	279
['KNN', 'RF', 'SVM', 'GNB', 'lightGBM', 'MLP']	0.8981	0.9342	0.9281	0.9311	0.7349	0.92877131	0.061	0.2934	0.1821	0.4635	280
['KNN', 'RF', 'LR', 'GNB', 'XGBoost', 'MLP']	0.8981	0.9342	0.9281	0.9311	0.7349	0.92877131	0.061	0.2934	0.1821	0.4635	281
['KNN', 'RF', 'LR', 'GNB', 'lightGBM', 'MLP']	0.8981	0.9342	0.9281	0.9311	0.7349	0.92877131	0.061	0.2934	0.1821	0.4635	282
['DT', 'RF', 'SVM', 'LR', 'GNB', 'XGBoost']	0.8981	0.9342	0.9281	0.9311	0.7349	0.92877131	0.061	0.2934	0.1821	0.4635	283
['DT', 'RF', 'SVM', 'LR', 'XGBoost', 'MLP']	0.8981	0.9342	0.9281	0.9311	0.7349	0.92877131	0.061	0.2934	0.1821	0.4635	284
['DT', 'RF', 'SVM', 'GNB', 'lightGBM', 'MLP']	0.8981	0.9342	0.9281	0.9311	0.7349	0.92877131	0.061	0.2934	0.1821	0.4635	285
['DT', 'RF', 'GNB', 'XGBoost', 'lightGBM', 'MLP']	0.8981	0.9342	0.9281	0.9311	0.7349	0.92877131	0.061	0.2934	0.1821	0.4635	286
['RF', 'SVM', 'LR', 'GNB', 'XGBoost', 'MLP']	0.8981	0.9342	0.9281	0.9311	0.7349	0.92877131	0.061	0.2934	0.1821	0.4635	287
['KNN', 'DT', 'RF', 'SVM', 'LR', 'GNB', 'MLP']	0.8981	0.9342	0.9281	0.9311	0.7349	0.92877131	0.061	0.2934	0.1821	0.4635	288
['KNN', 'DT', 'RF', 'SVM', 'LR', 'lightGBM', 'MLP']	0.8981	0.9342	0.9281	0.9311	0.7349	0.92877131	0.061	0.2934	0.1821	0.4635	289
['KNN', 'DT', 'RF', 'SVM', 'GNB', 'XGBoost', 'lightGBM']	0.8981	0.9342	0.9281	0.9311	0.7349	0.92877131	0.061	0.2934	0.1821	0.4635	290
['KNN', 'DT', 'RF', 'LR', 'GNB', 'XGBoost', 'MLP']	0.8981	0.9342	0.9281	0.9311	0.7349	0.92877131	0.061	0.2934	0.1821	0.4635	291
['KNN', 'DT', 'SVM', 'LR', 'XGBoost', 'lightGBM', 'MLP']	0.8981	0.9342	0.9281	0.9311	0.7349	0.92877131	0.061	0.2934	0.1821	0.4635	292
['KNN', 'RF', 'SVM', 'LR', 'GNB', 'XGBoost', 'MLP']	0.8981	0.9342	0.9281	0.9311	0.7349	0.92877131	0.061	0.2934	0.1821	0.4635	293
['KNN', 'RF', 'SVM', 'LR', 'XGBoost', 'lightGBM', 'MLP']	0.8981	0.9342	0.9281	0.9311	0.7349	0.92877131	0.061	0.2934	0.1821	0.4635	294
['KNN', 'DT', 'SVM', 'LR', 'GNB', 'XGBoost', 'lightGBM', 'MLP']	0.8981	0.9342	0.9281	0.9311	0.7349	0.92877131	0.061	0.2934	0.1821	0.4635	295
['KNN', 'DT']	0.8932	0.9172	0.9412	0.929	0.7135	0.9282469	0.061	0.2934	0.1821	0.4635	296
['KNN', 'MLP']	0.8932	0.9172	0.9412	0.929	0.7135	0.9282469	0.061	0.2934	0.1821	0.4635	297
['DT', 'SVM']	0.8932	0.9172	0.9412	0.929	0.7135	0.9282469	0.061	0.2934	0.1821	0.4635	298
['DT', 'LR']	0.8932	0.9172	0.9412	0.929	0.7135	0.9282469	0.061	0.2934	0.1821	0.4635	299
['SVM', 'XGBoost']	0.8932	0.9172	0.9412	0.929	0.7135	0.9282469	0.061	0.2934	0.1821	0.4635	300
['KNN', 'RF', 'XGBoost']	0.8932	0.9172	0.9412	0.929	0.7135	0.9282469	0.061	0.2934	0.1821	0.4635	301
['DT', 'SVM', 'XGBoost']	0.8932	0.9172	0.9412	0.929	0.7135	0.9282469	0.061	0.2934	0.1821	0.4635	302
['DT', 'GNB', 'XGBoost']	0.8932	0.9172	0.9412	0.929	0.7135	0.9282469	0.061	0.2934	0.1821	0.4635	303
['SVM', 'LR', 'lightGBM']	0.8932	0.9172	0.9412	0.929	0.7135	0.9282469	0.061	0.2934	0.1821	0.4635	304
['SVM', 'GNB', 'XGBoost']	0.8932	0.9172	0.9412	0.929	0.7135	0.9282469	0.061	0.2934	0.1821	0.4635	305
['KNN', 'DT', 'SVM', 'GNB']	0.8932	0.9172	0.9412	0.929	0.7135	0.9282469	0.061	0.2934	0.1821	0.4635	306
['KNN', 'DT', 'SVM', 'XGBoost']	0.8932	0.9172	0.9412	0.929	0.7135	0.9282469	0.061	0.2934	0.1821	0.4635	307
['KNN', 'SVM', 'GNB', 'XGBoost']	0.8932	0.9172	0.9412	0.929	0.7135	0.9282469	0.061	0.2934	0.1821	0.4635	308
['DT', 'SVM', 'GNB', 'XGBoost']	0.8932	0.9172	0.9412	0.929	0.7135	0.9282469	0.061	0.2934	0.1821	0.4635	309
['DT', 'RF', 'LR']	0.8981	0.94	0.9216	0.9307	0.7381	0.92773499	0.061	0.2934	0.1821	0.4635	310
['DT', 'RF', 'lightGBM', 'MLP']	0.8981	0.94	0.9216	0.9307	0.7381	0.92773499	0.061	0.2934	0.1821	0.4635	311
['KNN', 'DT', 'RF', 'GNB', 'XGBoost', 'MLP']	0.8981	0.94	0.9216	0.9307	0.7381	0.92773499	0.061	0.2934	0.1821	0.4635	312
['KNN', 'LR']	0.8932	0.9226	0.9346	0.9286	0.7171	0.9271084	0.061	0.2934	0.1821	0.4635	313
['KNN', 'LR', 'MLP']	0.8932	0.9226	0.9346	0.9286	0.7171	0.9271084	0.061	0.2934	0.1821	0.4635	314
['DT', 'SVM', 'GNB']	0.8932	0.9226	0.9346	0.9286	0.7171	0.9271084	0.061	0.2934	0.1821	0.4635	315
['SVM', 'XGBoost', 'MLP']	0.8932	0.9226	0.9346	0.9286	0.7171	0.9271084	0.061	0.2934	0.1821	0.4635	316
['XGBoost', 'lightGBM', 'MLP']	0.8932	0.9226	0.9346	0.9286	0.7171	0.9271084	0.061	0.2934	0.1821	0.4635	317
['KNN', 'DT', 'SVM', 'lightGBM']	0.8932	0.9226	0.9346	0.9286	0.7171	0.9271084	0.061	0.2934	0.1821	0.4635	318
['KNN', 'DT', 'XGBoost', 'lightGBM']	0.8932	0.9226	0.9346	0.9286	0.7171	0.9271084	0.061	0.2934	0.1821	0.4635	319
['KNN', 'SVM', 'LR', 'GNB']	0.8932	0.9226	0.9346	0.9286	0.7171	0.9271084	0.061	0.2934	0.1821	0.4635	320
['KNN', 'SVM', 'LR', 'MLP']	0.8932	0.9226	0.9346	0.9286	0.7171	0.9271084	0.061	0.2934	0.1821	0.4635	321
['KNN', 'SVM', 'GNB', 'lightGBM']	0.8932	0.9226	0.9346	0.9286	0.7171	0.9271084	0.061	0.2934	0.1821	0.4635	322
['KNN', 'SVM', 'XGBoost', 'lightGBM']	0.8932	0.9226	0.9346	0.9286	0.7171	0.9271084	0.061	0.2934	0.1821	0.4635	323
['SVM', 'GNB', 'XGBoost', 'lightGBM']	0.8932	0.9226	0.9346	0.9286	0.7171	0.9271084	0.061	0.2934	0.1821	0.4635	324
['KNN', 'RF', 'SVM', 'LR', 'MLP']	0.8932	0.9226	0.9346	0.9286	0.7171	0.9271084	0.061	0.2934	0.1821	0.4635	325
['KNN', 'SVM', 'GNB', 'XGBoost', 'lightGBM']	0.8932	0.9226	0.9346	0.9286	0.7171	0.9271084	0.061	0.2934	0.1821	0.4635	326
['KNN', 'DT', 'SVM', 'GNB', 'XGBoost', 'lightGBM']	0.8932	0.9226	0.9346	0.9286	0.7171	0.9271084	0.061	0.2934	0.1821	0.4635	327
['RF', 'GNB', 'XGBoost', 'MLP']	0.8981	0.9459	0.915	0.9302	0.7412	0.92664119	0.061	0.2934	0.1821	0.4635	328
['RF', 'LR']	0.8932	0.9281	0.9281	0.9281	0.7206	0.9259711	0.061	0.2934	0.1821	0.4635	329
['KNN', 'RF', 'LR']	0.8932	0.9281	0.9281	0.9281	0.7206	0.9259711	0.061	0.2934	0.1821	0.4635	330
['KNN', 'LR', 'lightGBM']	0.8932	0.9281	0.9281	0.9281	0.7206	0.9259711	0.061	0.2934	0.1821	0.4635	331
['RF', 'SVM', 'lightGBM']	0.8932	0.9281	0.9281	0.9281	0.7206	0.9259711	0.061	0.2934	0.1821	0.4635	332
['RF', 'XGBoost', 'lightGBM']	0.8932	0.9281	0.9281	0.9281	0.7206	0.9259711	0.061	0.2934	0.1821	0.4635	333
['KNN', 'RF', 'LR', 'MLP']	0.8932	0.9281	0.9281	0.9281	0.7206	0.9259711	0.061	0.2934	0.1821	0.4635	334
['KNN', 'RF', 'GNB', 'lightGBM']	0.8932	0.9281	0.9281	0.9281	0.7206	0.9259711	0.061	0.2934	0.1821	0.4635	335
['KNN', 'LR', 'GNB', 'XGBoost']	0.8932	0.9281	0.9281	0.9281	0.7206	0.9259711	0.061	0.2934	0.1821	0.4635	336
['RF', 'LR', 'GNB', 'MLP']	0.8932	0.9281	0.9281	0.9281	0.7206	0.9259711	0.061	0.2934	0.1821	0.4635	337
['KNN', 'DT', 'LR', 'GNB', 'lightGBM']	0.8932	0.9281	0.9281	0.9281	0.7206	0.9259711	0.061	0.2934	0.1821	0.4635	338
['KNN', 'RF', 'SVM', 'XGBoost', 'lightGBM']	0.8932	0.9281	0.9281	0.9281	0.7206	0.9259711	0.061	0.2934	0.1821	0.4635	339
['KNN', 'RF', 'LR', 'XGBoost', 'MLP']	0.8932	0.9281	0.9281	0.9281	0.7206	0.9259711	0.061	0.2934	0.1821	0.4635	340
['RF', 'SVM', 'LR', 'GNB', 'lightGBM']	0.8932	0.9281	0.9281	0.9281	0.7206	0.9259711	0.061	0.2934	0.1821	0.4635	341
['RF', 'SVM', 'LR', 'XGBoost', 'MLP']	0.8932	0.9281	0.9281	0.9281	0.7206	0.9259711	0.061	0.2934	0.1821	0.4635	342
['RF', 'LR', 'XGBoost', 'lightGBM', 'MLP']	0.8932	0.9281	0.9281	0.9281	0.7206	0.9259711	0.061	0.2934	0.1821	0.4635	343
['KNN', 'DT', 'SVM', 'LR', 'GNB', 'XGBoost']	0.8932	0.9281	0.9281	0.9281	0.7206	0.9259711	0.061	0.2934	0.1821	0.4635	344
['KNN', 'DT', 'SVM', 'LR', 'GNB', 'lightGBM']	0.8932	0.9281	0.9281	0.9281	0.7206	0.9259711	0.061	0.2934	0.1821	0.4635	345
['KNN', 'DT', 'SVM', 'GNB', 'lightGBM', 'MLP']	0.8932	0.9281	0.9281	0.9281	0.7206	0.9259711	0.061	0.2934	0.1821	0.4635	346
['KNN', 'DT', 'LR', 'GNB', 'XGBoost', 'lightGBM']	0.8932	0.9281	0.9281	0.9281	0.7206	0.9259711	0.061	0.2934	0.1821	0.4635	347
['KNN', 'DT', 'LR', 'GNB', 'lightGBM', 'MLP']	0.8932	0.9281	0.9281	0.9281	0.7206	0.9259711	0.061	0.2934	0.1821	0.4635	348
['KNN', 'DT', 'SVM', 'LR', 'GNB', 'XGBoost', 'lightGBM']	0.8932	0.9281	0.9281	0.9281	0.7206	0.9259711	0.061	0.2934	0.1821	0.4635	349
['KNN', 'DT', 'SVM', 'LR', 'GNB', 'lightGBM', 'MLP']	0.8932	0.9281	0.9281	0.9281	0.7206	0.9259711	0.061	0.2934	0.1821	0.4635	350
['KNN', 'DT', 'LR', 'GNB', 'XGBoost', 'lightGBM', 'MLP']	0.8932	0.9281	0.9281	0.9281	0.7206	0.9259711	0.061	0.2934	0.1821	0.4635	351
['KNN', 'RF', 'SVM', 'GNB', 'XGBoost', 'lightGBM', 'MLP']	0.8932	0.9281	0.9281	0.9281	0.7206	0.9259711	0.061	0.2934	0.1821	0.4635	352
['KNN', 'RF', 'LR', 'GNB', 'XGBoost', 'lightGBM', 'MLP']	0.8932	0.9281	0.9281	0.9281	0.7206	0.9259711	0.061	0.2934	0.1821	0.4635	353
['DT', 'RF', 'LR', 'GNB', 'XGBoost', 'lightGBM', 'MLP']	0.8932	0.9281	0.9281	0.9281	0.7206	0.9259711	0.061	0.2934	0.1821	0.4635	354
['KNN', 'RF', 'SVM', 'LR', 'GNB', 'XGBoost', 'lightGBM', 'MLP']	0.8932	0.9281	0.9281	0.9281	0.7206	0.9259711	0.061	0.2934	0.1821	0.4635	355
['KNN', 'SVM']	0.8883	0.9114	0.9412	0.926	0.6986	0.92550132	0.061	0.2934	0.1821	0.4635	356
['KNN', 'DT', 'GNB', 'XGBoost']	0.8883	0.9114	0.9412	0.926	0.6986	0.92550132	0.061	0.2934	0.1821	0.4635	357

['KNN', 'DT', 'SVM', 'GNB', 'XGBoost']	0.8883	0.9114	0.9412	0.926	0.6986	0.92550132	0.061	0.2934	0.1821	0.4635	358
['KNN', 'DT', 'LR']	0.8932	0.9338	0.9216	0.9276	0.724	0.92487022	0.061	0.2934	0.1821	0.4635	359
['KNN', 'LR', 'GNB']	0.8932	0.9338	0.9216	0.9276	0.724	0.92487022	0.061	0.2934	0.1821	0.4635	360
['DT', 'RF', 'GNB']	0.8932	0.9338	0.9216	0.9276	0.724	0.92487022	0.061	0.2934	0.1821	0.4635	361
['RF', 'LR', 'lightGBM', 'MLP']	0.8932	0.9338	0.9216	0.9276	0.724	0.92487022	0.061	0.2934	0.1821	0.4635	362
['KNN', 'DT', 'RF', 'SVM', 'lightGBM']	0.8932	0.9338	0.9216	0.9276	0.724	0.92487022	0.061	0.2934	0.1821	0.4635	363
['DT', 'RF', 'GNB', 'XGBoost', 'lightGBM']	0.8932	0.9338	0.9216	0.9276	0.724	0.92487022	0.061	0.2934	0.1821	0.4635	364
['KNN', 'DT', 'SVM', 'LR', 'GNB', 'MLP']	0.8932	0.9338	0.9216	0.9276	0.724	0.92487022	0.061	0.2934	0.1821	0.4635	365
['KNN', 'RF', 'SVM', 'LR', 'GNB', 'XGBoost']	0.8932	0.9338	0.9216	0.9276	0.724	0.92487022	0.061	0.2934	0.1821	0.4635	366
['DT', 'RF', 'SVM', 'GNB', 'XGBoost', 'lightGBM']	0.8932	0.9338	0.9216	0.9276	0.724	0.92487022	0.061	0.2934	0.1821	0.4635	367
['DT', 'RF', 'SVM', 'GNB', 'XGBoost', 'MLP']	0.8932	0.9338	0.9216	0.9276	0.724	0.92487022	0.061	0.2934	0.1821	0.4635	368
['KNN', 'RF']	0.8883	0.9167	0.9346	0.9256	0.7023	0.92434461	0.061	0.2934	0.1821	0.4635	369
['KNN', 'XGBoost']	0.8883	0.9167	0.9346	0.9256	0.7023	0.92434461	0.061	0.2934	0.1821	0.4635	370
['KNN', 'lightGBM']	0.8883	0.9167	0.9346	0.9256	0.7023	0.92434461	0.061	0.2934	0.1821	0.4635	371
['SVM', 'LR']	0.8883	0.9167	0.9346	0.9256	0.7023	0.92434461	0.061	0.2934	0.1821	0.4635	372
['SVM', 'lightGBM']	0.8883	0.9167	0.9346	0.9256	0.7023	0.92434461	0.061	0.2934	0.1821	0.4635	373
['XGBoost', 'MLP']	0.8883	0.9167	0.9346	0.9256	0.7023	0.92434461	0.061	0.2934	0.1821	0.4635	374
['KNN', 'RF', 'lightGBM']	0.8883	0.9167	0.9346	0.9256	0.7023	0.92434461	0.061	0.2934	0.1821	0.4635	375
['DT', 'SVM', 'LR']	0.8883	0.9167	0.9346	0.9256	0.7023	0.92434461	0.061	0.2934	0.1821	0.4635	376
['DT', 'SVM', 'MLP']	0.8883	0.9167	0.9346	0.9256	0.7023	0.92434461	0.061	0.2934	0.1821	0.4635	377
['SVM', 'LR', 'XGBoost']	0.8883	0.9167	0.9346	0.9256	0.7023	0.92434461	0.061	0.2934	0.1821	0.4635	378
['SVM', 'LR', 'MLP']	0.8883	0.9167	0.9346	0.9256	0.7023	0.92434461	0.061	0.2934	0.1821	0.4635	379
['SVM', 'XGBoost', 'lightGBM']	0.8883	0.9167	0.9346	0.9256	0.7023	0.92434461	0.061	0.2934	0.1821	0.4635	380
['KNN', 'GNB', 'XGBoost', 'lightGBM']	0.8883	0.9167	0.9346	0.9256	0.7023	0.92434461	0.061	0.2934	0.1821	0.4635	381
['DT', 'SVM', 'XGBoost', 'lightGBM']	0.8883	0.9167	0.9346	0.9256	0.7023	0.92434461	0.061	0.2934	0.1821	0.4635	382
['SVM', 'GNB', 'XGBoost', 'MLP']	0.8883	0.9167	0.9346	0.9256	0.7023	0.92434461	0.061	0.2934	0.1821	0.4635	383
['SVM', 'XGBoost', 'lightGBM', 'MLP']	0.8883	0.9167	0.9346	0.9256	0.7023	0.92434461	0.061	0.2934	0.1821	0.4635	384
['KNN', 'DT', 'SVM', 'GNB', 'lightGBM']	0.8883	0.9167	0.9346	0.9256	0.7023	0.92434461	0.061	0.2934	0.1821	0.4635	385
['KNN', 'DT', 'SVM', 'XGBoost', 'lightGBM']	0.8883	0.9167	0.9346	0.9256	0.7023	0.92434461	0.061	0.2934	0.1821	0.4635	386
['LR', 'XGBoost']	0.8883	0.9221	0.9281	0.9251	0.706	0.9231891	0.061	0.2934	0.1821	0.4635	387
['KNN', 'DT', 'XGBoost']	0.8883	0.9221	0.9281	0.9251	0.706	0.9231891	0.061	0.2934	0.1821	0.4635	388
['KNN', 'DT', 'lightGBM']	0.8883	0.9221	0.9281	0.9251	0.706	0.9231891	0.061	0.2934	0.1821	0.4635	389
['DT', 'XGBoost', 'lightGBM']	0.8883	0.9221	0.9281	0.9251	0.706	0.9231891	0.061	0.2934	0.1821	0.4635	390
['RF', 'SVM', 'LR']	0.8883	0.9221	0.9281	0.9251	0.706	0.9231891	0.061	0.2934	0.1821	0.4635	391
['RF', 'SVM', 'GNB']	0.8883	0.9221	0.9281	0.9251	0.706	0.9231891	0.061	0.2934	0.1821	0.4635	392
['SVM', 'GNB', 'lightGBM']	0.8883	0.9221	0.9281	0.9251	0.706	0.9231891	0.061	0.2934	0.1821	0.4635	393
['KNN', 'SVM', 'GNB', 'MLP']	0.8883	0.9221	0.9281	0.9251	0.706	0.9231891	0.061	0.2934	0.1821	0.4635	394
['DT', 'GNB', 'XGBoost', 'MLP']	0.8883	0.9221	0.9281	0.9251	0.706	0.9231891	0.061	0.2934	0.1821	0.4635	395
['SVM', 'LR', 'GNB', 'XGBoost']	0.8883	0.9221	0.9281	0.9251	0.706	0.9231891	0.061	0.2934	0.1821	0.4635	396
['LR', 'XGBoost', 'lightGBM', 'MLP']	0.8883	0.9221	0.9281	0.9251	0.706	0.9231891	0.061	0.2934	0.1821	0.4635	397
['KNN', 'RF', 'SVM', 'LR', 'XGBoost']	0.8883	0.9221	0.9281	0.9251	0.706	0.9231891	0.061	0.2934	0.1821	0.4635	398
['SVM', 'LR', 'XGBoost', 'lightGBM', 'MLP']	0.8883	0.9221	0.9281	0.9251	0.706	0.9231891	0.061	0.2934	0.1821	0.4635	399
['MLP']	0.8835	0.9057	0.9412	0.9231	0.6834	0.9228264	0.061	0.2934	0.1821	0.4635	400
['KNN', 'DT', 'GNB']	0.8835	0.9057	0.9412	0.9231	0.6834	0.9228264	0.061	0.2934	0.1821	0.4635	401
['KNN', 'SVM', 'GNB']	0.8835	0.9057	0.9412	0.9231	0.6834	0.9228264	0.061	0.2934	0.1821	0.4635	402
['GNB', 'lightGBM']	0.8883	0.9276	0.9216	0.9246	0.7096	0.9220518	0.061	0.2934	0.1821	0.4635	403
['DT', 'LR', 'GNB']	0.8883	0.9276	0.9216	0.9246	0.7096	0.9220518	0.061	0.2934	0.1821	0.4635	404
['DT', 'GNB', 'lightGBM']	0.8883	0.9276	0.9216	0.9246	0.7096	0.9220518	0.061	0.2934	0.1821	0.4635	405
['DT', 'GNB', 'MLP']	0.8883	0.9276	0.9216	0.9246	0.7096	0.9220518	0.061	0.2934	0.1821	0.4635	406
['GNB', 'XGBoost', 'lightGBM']	0.8883	0.9276	0.9216	0.9246	0.7096	0.9220518	0.061	0.2934	0.1821	0.4635	407
['GNB', 'lightGBM', 'MLP']	0.8883	0.9276	0.9216	0.9246	0.7096	0.9220518	0.061	0.2934	0.1821	0.4635	408
['KNN', 'DT', 'LR', 'GNB']	0.8883	0.9276	0.9216	0.9246	0.7096	0.9220518	0.061	0.2934	0.1821	0.4635	409
['DT', 'SVM', 'GNB', 'lightGBM']	0.8883	0.9276	0.9216	0.9246	0.7096	0.9220518	0.061	0.2934	0.1821	0.4635	410
['DT', 'GNB', 'XGBoost', 'lightGBM']	0.8883	0.9276	0.9216	0.9246	0.7096	0.9220518	0.061	0.2934	0.1821	0.4635	411
['SVM', 'GNB', 'lightGBM', 'MLP']	0.8883	0.9276	0.9216	0.9246	0.7096	0.9220518	0.061	0.2934	0.1821	0.4635	412
['LR', 'GNB', 'lightGBM', 'MLP']	0.8883	0.9276	0.9216	0.9246	0.7096	0.9220518	0.061	0.2934	0.1821	0.4635	413
['KNN', 'DT', 'RF', 'XGBoost', 'lightGBM']	0.8883	0.9276	0.9216	0.9246	0.7096	0.9220518	0.061	0.2934	0.1821	0.4635	414
['DT', 'SVM', 'GNB', 'XGBoost', 'lightGBM']	0.8883	0.9276	0.9216	0.9246	0.7096	0.9220518	0.061	0.2934	0.1821	0.4635	415
['SVM', 'LR', 'GNB', 'lightGBM', 'MLP']	0.8883	0.9276	0.9216	0.9246	0.7096	0.9220518	0.061	0.2934	0.1821	0.4635	416
['LR']	0.8835	0.9108	0.9346	0.9226	0.6874	0.92158692	0.061	0.2934	0.1821	0.4635	417
['DT', 'XGBoost']	0.8835	0.9108	0.9346	0.9226	0.6874	0.92158692	0.061	0.2934	0.1821	0.4635	418
['DT', 'MLP']	0.8835	0.9108	0.9346	0.9226	0.6874	0.92158692	0.061	0.2934	0.1821	0.4635	419
['RF', 'GNB']	0.8883	0.9333	0.915	0.9241	0.7132	0.92092158	0.061	0.2934	0.1821	0.4635	420
['KNN', 'RF', 'GNB']	0.8883	0.9333	0.915	0.9241	0.7132	0.92092158	0.061	0.2934	0.1821	0.4635	421
['KNN', 'RF', 'LR', 'lightGBM']	0.8883	0.9333	0.915	0.9241	0.7132	0.92092158	0.061	0.2934	0.1821	0.4635	422
['KNN', 'GNB', 'lightGBM']	0.8835	0.9161	0.9281	0.9221	0.6913	0.9204132	0.061	0.2934	0.1821	0.4635	423
['KNN', 'XGBoost', 'lightGBM']	0.8835	0.9161	0.9281	0.9221	0.6913	0.9204132	0.061	0.2934	0.1821	0.4635	424
['DT', 'LR', 'MLP']	0.8835	0.9161	0.9281	0.9221	0.6913	0.9204132	0.061	0.2934	0.1821	0.4635	425
['DT', 'XGBoost', 'MLP']	0.8835	0.9161	0.9281	0.9221	0.6913	0.9204132	0.061	0.2934	0.1821	0.4635	426
['SVM', 'lightGBM', 'MLP']	0.8835	0.9161	0.9281	0.9221	0.6913	0.9204132	0.061	0.2934	0.1821	0.4635	427
['LR', 'XGBoost', 'lightGBM']	0.8835	0.9161	0.9281	0.9221	0.6913	0.9204132	0.061	0.2934	0.1821	0.4635	428
['LR', 'XGBoost', 'MLP']	0.8835	0.9161	0.9281	0.9221	0.6913	0.9204132	0.061	0.2934	0.1821	0.4635	429
['LR', 'lightGBM', 'MLP']	0.8835	0.9161	0.9281	0.9221	0.6913	0.9204132	0.061	0.2934	0.1821	0.4635	430
['GNB', 'XGBoost', 'MLP']	0.8835	0.9161	0.9281	0.9221	0.6913	0.9204132	0.061	0.2934	0.1821	0.4635	431
['DT', 'SVM', 'XGBoost', 'MLP']	0.8835	0.9161	0.9281	0.9221	0.6913	0.9204132	0.061	0.2934	0.1821	0.4635	432
['DT', 'LR', 'XGBoost', 'MLP']	0.8835	0.9161	0.9281	0.9221	0.6913	0.9204132	0.061	0.2934	0.1821	0.4635	433
['SVM', 'LR', 'XGBoost', 'lightGBM']	0.8835	0.9161	0.9281	0.9221	0.6913	0.9204132	0.061	0.2934	0.1821	0.4635	434
['SVM', 'LR', 'XGBoost', 'MLP']	0.8835	0.9161	0.9281	0.9221	0.6913	0.9204132	0.061	0.2934	0.1821	0.4635	435
['DT', 'SVM', 'LR', 'XGBoost', 'MLP']	0.8835	0.9161	0.9281	0.9221	0.6913	0.9204132	0.061	0.2934	0.1821	0.4635	436
['DT', 'SVM', 'GNB', 'XGBoost', 'MLP']	0.8835	0.9161	0.9281	0.9221	0.6913	0.9204132	0.061	0.2934	0.1821	0.4635	437
['SVM', 'LR', 'GNB', 'XGBoost', 'lightGBM']	0.8835	0.9161	0.9281	0.9221	0.6913	0.9204132	0.061	0.2934	0.1821	0.4635	438
['XGBoost', 'lightGBM']	0.8835	0.9216	0.9216	0.9216	0.6952	0.9192759	0.061	0.2934	0.1821	0.4635	439
['lightGBM', 'MLP']	0.8835	0.9216	0.9216	0.9216	0.6952	0.9192759	0.061	0.2934	0.1821	0.4635	440
['DT', 'lightGBM', 'MLP']	0.8835	0.9216	0.9216	0.9216	0.6952	0.9192759	0.061	0.2934	0.1821	0.4635	441
['KNN', 'RF', 'GNB', 'XGBoost']	0.8835	0.9216	0.9216	0.9216	0.6952	0.9192759	0.061	0.2934	0.1821	0.4635	442
['KNN', 'RF', 'XGBoost', 'lightGBM']	0.8835	0.9216	0.9216	0.9216	0.6952	0.9192759	0.061	0.2934	0.1821	0.4635	443
['DT', 'SVM', 'LR', 'GNB']	0.8835	0.9216	0.9216	0.9216	0.6952	0.9192759	0.061	0.2934	0.1821	0.4635	444
['DT', 'SVM', 'GNB', 'MLP']	0.8835	0.9216	0.9216	0.9216	0.6952	0.9192759	0.061	0.2934	0.1821	0.4635	445
['DT', 'SVM', 'lightGBM', 'MLP']	0.8835	0.9216	0.9216	0.9216	0.6952	0.9192759	0.061	0.2934	0.1821	0.4635	446
['DT', 'LR', 'GNB', 'XGBoost']	0.8835	0.9216	0.9216	0.9216	0.6952	0.9192759	0.061	0.2934	0.1821	0.4635	447

['DT', 'LR', 'GNB', 'MLP']	0.8835	0.9216	0.9216	0.9216	0.6952	0.9192759	0.061	0.2934	0.1821	0.4635	448
['DT', 'LR', 'lightGBM', 'MLP']	0.8835	0.9216	0.9216	0.9216	0.6952	0.9192759	0.061	0.2934	0.1821	0.4635	449
['DT', 'GNB', 'lightGBM', 'MLP']	0.8835	0.9216	0.9216	0.9216	0.6952	0.9192759	0.061	0.2934	0.1821	0.4635	450
['DT', 'XGBoost', 'lightGBM', 'MLP']	0.8835	0.9216	0.9216	0.9216	0.6952	0.9192759	0.061	0.2934	0.1821	0.4635	451
['LR', 'GNB', 'XGBoost', 'lightGBM']	0.8835	0.9216	0.9216	0.9216	0.6952	0.9192759	0.061	0.2934	0.1821	0.4635	452
['GNB', 'XGBoost', 'lightGBM', 'MLP']	0.8835	0.9216	0.9216	0.9216	0.6952	0.9192759	0.061	0.2934	0.1821	0.4635	453
['DT', 'SVM', 'LR', 'GNB', 'MLP']	0.8835	0.9216	0.9216	0.9216	0.6952	0.9192759	0.061	0.2934	0.1821	0.4635	454
['DT', 'SVM', 'LR', 'lightGBM', 'MLP']	0.8835	0.9216	0.9216	0.9216	0.6952	0.9192759	0.061	0.2934	0.1821	0.4635	455
['DT', 'SVM', 'GNB', 'lightGBM', 'MLP']	0.8835	0.9216	0.9216	0.9216	0.6952	0.9192759	0.061	0.2934	0.1821	0.4635	456
['DT', 'SVM', 'XGBoost', 'lightGBM', 'MLP']	0.8835	0.9216	0.9216	0.9216	0.6952	0.9192759	0.061	0.2934	0.1821	0.4635	457
['DT', 'LR', 'GNB', 'lightGBM', 'MLP']	0.8835	0.9216	0.9216	0.9216	0.6952	0.9192759	0.061	0.2934	0.1821	0.4635	458
['DT', 'LR', 'XGBoost', 'lightGBM', 'MLP']	0.8835	0.9216	0.9216	0.9216	0.6952	0.9192759	0.061	0.2934	0.1821	0.4635	459
['DT', 'GNB', 'XGBoost', 'lightGBM', 'MLP']	0.8835	0.9216	0.9216	0.9216	0.6952	0.9192759	0.061	0.2934	0.1821	0.4635	460
['SVM', 'GNB', 'XGBoost', 'lightGBM', 'MLP']	0.8835	0.9216	0.9216	0.9216	0.6952	0.9192759	0.061	0.2934	0.1821	0.4635	461
['DT', 'SVM', 'LR', 'GNB', 'XGBoost', 'lightGBM']	0.8835	0.9216	0.9216	0.9216	0.6952	0.9192759	0.061	0.2934	0.1821	0.4635	462
['DT', 'SVM', 'LR', 'XGBoost', 'lightGBM', 'MLP']	0.8835	0.9216	0.9216	0.9216	0.6952	0.9192759	0.061	0.2934	0.1821	0.4635	463
['DT', 'SVM', 'GNB', 'XGBoost', 'lightGBM', 'MLP']	0.8835	0.9216	0.9216	0.9216	0.6952	0.9192759	0.061	0.2934	0.1821	0.4635	464
['DT', 'LR', 'GNB', 'XGBoost', 'lightGBM', 'MLP']	0.8835	0.9216	0.9216	0.9216	0.6952	0.9192759	0.061	0.2934	0.1821	0.4635	465
['DT', 'SVM', 'LR', 'GNB', 'XGBoost', 'lightGBM', 'MLP']	0.8835	0.9216	0.9216	0.9216	0.6952	0.9192759	0.061	0.2934	0.1821	0.4635	466
['XGBoost']	0.8786	0.9051	0.9346	0.9196	0.6724	0.91885955	0.061	0.2934	0.1821	0.4635	467
['LR', 'GNB', 'lightGBM']	0.8835	0.9272	0.915	0.9211	0.6989	0.91812747	0.061	0.2934	0.1821	0.4635	468
['KNN', 'DT', 'RF', 'GNB']	0.8835	0.9272	0.915	0.9211	0.6989	0.91812747	0.061	0.2934	0.1821	0.4635	469
['DT', 'LR', 'GNB', 'lightGBM']	0.8835	0.9272	0.915	0.9211	0.6989	0.91812747	0.061	0.2934	0.1821	0.4635	470
['DT', 'SVM', 'LR', 'GNB', 'lightGBM']	0.8835	0.9272	0.915	0.9211	0.6989	0.91812747	0.061	0.2934	0.1821	0.4635	471
['LR', 'MLP']	0.8786	0.9103	0.9281	0.9191	0.6765	0.91766762	0.061	0.2934	0.1821	0.4635	472
['KNN', 'SVM', 'XGBoost']	0.8786	0.9103	0.9281	0.9191	0.6765	0.91766762	0.061	0.2934	0.1821	0.4635	473
['KNN', 'GNB', 'XGBoost']	0.8786	0.9103	0.9281	0.9191	0.6765	0.91766762	0.061	0.2934	0.1821	0.4635	474
['DT', 'SVM', 'LR', 'MLP']	0.8786	0.9103	0.9281	0.9191	0.6765	0.91766762	0.061	0.2934	0.1821	0.4635	475
['KNN', 'DT', 'GNB', 'XGBoost', 'lightGBM']	0.8786	0.9103	0.9281	0.9191	0.6765	0.91766762	0.061	0.2934	0.1821	0.4635	476
['DT', 'RF', 'SVM', 'LR', 'lightGBM']	0.8835	0.9329	0.9085	0.9205	0.7025	0.91698024	0.061	0.2934	0.1821	0.4635	477
['DT', 'SVM', 'lightGBM']	0.8786	0.9156	0.9216	0.9186	0.6805	0.9164939	0.061	0.2934	0.1821	0.4635	478
['LR', 'GNB', 'XGBoost']	0.8786	0.9156	0.9216	0.9186	0.6805	0.9164939	0.061	0.2934	0.1821	0.4635	479
['DT', 'SVM', 'LR', 'XGBoost']	0.8786	0.9156	0.9216	0.9186	0.6805	0.9164939	0.061	0.2934	0.1821	0.4635	480
['DT', 'SVM', 'LR', 'lightGBM']	0.8786	0.9156	0.9216	0.9186	0.6805	0.9164939	0.061	0.2934	0.1821	0.4635	481
['SVM', 'LR', 'lightGBM', 'MLP']	0.8786	0.9156	0.9216	0.9186	0.6805	0.9164939	0.061	0.2934	0.1821	0.4635	482
['DT', 'SVM', 'LR', 'GNB', 'XGBoost']	0.8786	0.9156	0.9216	0.9186	0.6805	0.9164939	0.061	0.2934	0.1821	0.4635	483
['DT', 'LR', 'GNB', 'XGBoost', 'MLP']	0.8786	0.9156	0.9216	0.9186	0.6805	0.9164939	0.061	0.2934	0.1821	0.4635	484
['LR', 'GNB', 'XGBoost', 'lightGBM', 'MLP']	0.8786	0.9156	0.9216	0.9186	0.6805	0.9164939	0.061	0.2934	0.1821	0.4635	485
['DT', 'SVM', 'LR', 'GNB', 'XGBoost', 'MLP']	0.8786	0.9156	0.9216	0.9186	0.6805	0.9164939	0.061	0.2934	0.1821	0.4635	486
['SVM', 'LR', 'GNB', 'XGBoost', 'lightGBM', 'MLP']	0.8786	0.9156	0.9216	0.9186	0.6805	0.9164939	0.061	0.2934	0.1821	0.4635	487
['KNN']	0.8786	0.9211	0.915	0.918	0.6844	0.91528091	0.061	0.2934	0.1821	0.4635	488
['lightGBM']	0.8786	0.9211	0.915	0.918	0.6844	0.91528091	0.061	0.2934	0.1821	0.4635	489
['DT', 'GNB']	0.8786	0.9211	0.915	0.918	0.6844	0.91528091	0.061	0.2934	0.1821	0.4635	490
['DT', 'lightGBM']	0.8786	0.9211	0.915	0.918	0.6844	0.91528091	0.061	0.2934	0.1821	0.4635	491
['DT', 'LR', 'lightGBM']	0.8786	0.9211	0.915	0.918	0.6844	0.91528091	0.061	0.2934	0.1821	0.4635	492
['DT', 'LR', 'XGBoost', 'lightGBM']	0.8786	0.9211	0.915	0.918	0.6844	0.91528091	0.061	0.2934	0.1821	0.4635	493
['SVM', 'LR', 'GNB', 'lightGBM']	0.8786	0.9211	0.915	0.918	0.6844	0.91528091	0.061	0.2934	0.1821	0.4635	494
['DT', 'LR', 'GNB', 'XGBoost', 'lightGBM']	0.8786	0.9211	0.915	0.918	0.6844	0.91528091	0.061	0.2934	0.1821	0.4635	495
['DT', 'SVM', 'LR', 'GNB', 'lightGBM', 'MLP']	0.8786	0.9211	0.915	0.918	0.6844	0.91528091	0.061	0.2934	0.1821	0.4635	496
['KNN', 'DT', 'GNB', 'lightGBM']	0.8738	0.9097	0.9216	0.9156	0.6656	0.91373621	0.061	0.2934	0.1821	0.4635	497
['LR', 'GNB', 'XGBoost', 'MLP']	0.8738	0.9097	0.9216	0.9156	0.6656	0.91373621	0.061	0.2934	0.1821	0.4635	498
['SVM', 'LR', 'GNB', 'XGBoost', 'MLP']	0.8738	0.9097	0.9216	0.9156	0.6656	0.91373621	0.061	0.2934	0.1821	0.4635	499
['DT', 'LR', 'XGBoost']	0.8738	0.915	0.915	0.915	0.6697	0.9124868	0.061	0.2934	0.1821	0.4635	500
['SVM', 'LR', 'GNB']	0.8738	0.915	0.915	0.915	0.6697	0.9124868	0.061	0.2934	0.1821	0.4635	501
['SVM', 'GNB', 'MLP']	0.8738	0.915	0.915	0.915	0.6697	0.9124868	0.061	0.2934	0.1821	0.4635	502
['SVM', 'LR', 'GNB', 'MLP']	0.8738	0.915	0.915	0.915	0.6697	0.9124868	0.061	0.2934	0.1821	0.4635	503
['DT', 'SVM', 'LR', 'XGBoost', 'lightGBM']	0.8738	0.915	0.915	0.915	0.6697	0.9124868	0.061	0.2934	0.1821	0.4635	504
['DT']	0.8689	0.9145	0.9085	0.9115	0.6591	0.9085675	0.061	0.2934	0.1821	0.4635	505
['LR', 'GNB']	0.8689	0.9145	0.9085	0.9115	0.6591	0.9085675	0.061	0.2934	0.1821	0.4635	506
['GNB', 'MLP']	0.8689	0.9145	0.9085	0.9115	0.6591	0.9085675	0.061	0.2934	0.1821	0.4635	507
['LR', 'GNB', 'MLP']	0.8641	0.9139	0.902	0.9079	0.6487	0.90458974	0.061	0.2934	0.1821	0.4635	508
['KNN', 'GNB']	0.8495	0.9122	0.8824	0.897	0.618	0.89258678	0.061	0.2934	0.1821	0.4635	509
['GNB']	0.8204	0.9028	0.8497	0.8754	0.5547	0.86949416	0.061	0.2934	0.1821	0.4635	510
